# The repetition of errors in recall: a review of four ‘fragmentation’ experiments

**DOI:** 10.1007/s00426-021-01598-z

**Published:** 2022-03-14

**Authors:** Donald Laming

**Affiliations:** Department of Psychology, University of Cambridge, Downing Street, Cambridge, CB2 3EB England, UK

## Abstract

This review reanalyses the data from four experiments originally designed to test the fragmentation hypothesis. Participants were asked to recall triple or quadruple associates, cued by each of their components in turn, and to guess if they could not remember. There were many errors in recall and many of those errors were repetitions of previous errors. This reanalysis focuses, not on the fragmentation hypothesis, but on the repetition of errors. It works backwards through sequences of test trials to discover the best prior match to the responses on each trial. It reports frequencies of different categories of repetition, conditional probabilities of repetition, correct recalls, and the probability of repetition in relation to the lag between trial and match in the test sequence. These results may be summarised as (1) every event (a stimulus or a response or just a retrieval) to which the participant attends is separately recorded in memory, creating an ordered record of those events that have engaged the participant’s attention; (2) the compilation of the record is automatic; while attention to a stimulus is at the participant’s disposal, the consequent entry into memory is not, and (3) the retrieval of a potential response from memory is spontaneous; that retrieval becomes an overt response if it is compatible with the cue. This makes sense of a number of historic anomalies in the study of recall and informs some contemporary problems in the study of short-term memory.

**Supplementary Information:**

The online version contains supplementary material available at 10.1007/s00426-021-01598-z.

## Introduction

This review reanalyses the data from four experiments on memory for stimuli comprised of three or four distinct components. Recall was tested by presenting a single component as cue and asking: What went with it? Each individual component was presented in turn as cue, but separated, of course, by tests of other stimuli. In three of these experiments participants were asked to guess if they could not remember. Many of those guesses turned out to be repetitions, in whole or in part, of the cue and responses on some previous test trial.

**Fig. 1:**
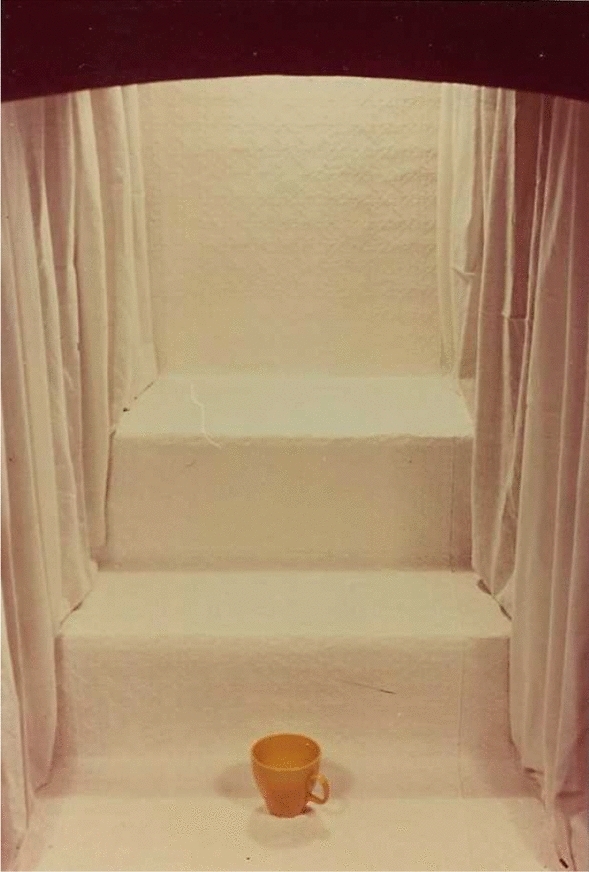
Sample stimulus from Jones (1978) ( I thank Greg Jones for the photograph reproduced as Fig. 1 and for an electronic copy of his original data.)

Figure 1 reproduces a sample stimulus from Jones (1978). There is a **yellow cup** located in the **centre** of the **bottom** shelf in a simulated shop window. Viewing the complete collection of stimuli, that cup might have been any one of nine **Objects** (ball, cup, comb, [toy] flute, fork, [toy] gun, milk bottle, scissors, or a screwdriver). It might have been painted in any one of nine **Colours** (black, blue, brown, green, grey, orange, pink, red, yellow), and might have been placed in any one of nine **Locations** (left, centre or right on the top, middle or bottom shelf in the window). Participants were shown a set of nine stimuli, so constructed that each attribute value (**Colour, Location, Object**) appeared exactly once in the set (and different sets, of course, combined the attribute values differently). Those nine stimuli had to be presented in some order and **Serial position** in that presentation was used as a fourth attribute, both as cue and as component to be recalled.

The stimuli were presented on slides in a Kodak carousel projector for 2.2 s each. After counting backwards by threes for 25 s from some large three-digit number, there followed 36 trials, each represented by a cue on a separate page of a booklet. Each page contained one of the attribute values and asked what other values had been combined with it. So, **yellow** as cue asked for *cup, bottom centre,* and *6*th; while **6** asked for *yellow, cup, bottom centre*. Each trial comprised a cue and three responses. The different stimuli were cued every nine trials and participants were asked to guess if they could not recall. Most of those ‘guesses’ turned out to be repetitions, in whole or in part, of the combination of cue and responses on some previous trial, and analysis of those repetitions provides a means of observing the storage and retrieval of memories over the space of 20 min.

There were 36 participants who each viewed three different sets of nine stimuli, each followed by 36 test trials, in the space of an hour. Each set of stimuli was presented and tested, and the participants rested, before the next was presented. Thirty-six participants, three sets of stimuli and 36 test trials give a total of 3888 test trials.

Table 1 reproduces the data from one presentation set by one participant to show, first, that many sets of responses are repetitions, in whole or in part, of some previous trial and, second, to illustrate the variety of those repetitions. Trials 1–9 are the stimuli; Trials 10–45 are test trials. Each stimulus has four *attributes*, **Colour, Location, Object** and **Sequential position**. Column 2 is the cue attribute, which changes with each trial, and Columns 3–6 the combination of cue and responses actually recorded. Columns 9–12 reproduce the content of the preceding stimulus or trial that best matches these responses (the most probable *source*). Column 7 is the trial number of that source (less than 10 indicates a stimulus) and Column 8 the nature of the match. C, L, O and S indicate correct responses, so CLOS means that all responses are correct (Trials 21, 31). ‘_’ indicates an error, so CLO_ indicates a triple match with a stimulus (Trials 16, 20 22, 40); likewise, CL_S, C_OS and _LOS mean that two responses are correct, but the third is wrong; and CL__ etc. means only one response is correct.

**Table 1:** Sample data from one presentation set by one participant in Jones (1978). Trials 1–9 are the stimuli; Trials 10–45 are test trials

Trial	Cue	Cue and observed responses				Source	Retrieval	Components of presumed source			
		Colour	Location	Object	Seq post’n			Colour	Location	Object	Seq post’n
1		Pink	Top_cent	Scissors							
2		Grey	Top_rt	Sc’driver							
3		Blue	Mid_cent	Gun							
4		Yellow	Mid_rt	M’kb’tle							
5		Orange	Mid_lft	Comb							
6		Black	Btm_cent	Fork							
7		Green	Top_lft	Cup							
8		Red	Btm_lft	Ball							
9		Brown	Btm_rt	Flute							
10	C	Brown	Mid_lft	Comb	7	5	ygs(_LO_)	Orange	Mid_lft	Comb	5
11	O	Yellow	Mid_cent	M’kb’tle	4	4	C_OS	Yellow	Mid_rt	M’kb’tle	4
12	L	Brown	Top_rt	Gun	3	3	ygs(__OS)	Blue	Mid_cent	Gun	3
13	S	Red	Btm_cent	Ball	5	8	ygs(C_O_)	Red	Btm_lft	Ball	8
14	O	Red	Btm_cent	Ball	6	13	crc(CLO_)	Red	Btm_cent	Ball	5
15	L	Grey	Top_lft	Flute	3		Null				
16	C	Black	Btm_cent	Fork	7	6	CLO_	Black	Btm_cent	Fork	6
17	O	Green	Mid_cent	Scissors	4	11	yrc(_L_S)	Yellow	Mid_cent	M’kb’tle	4
18	S	Grey	Mid_cent	Gun	3	3	LOS	Blue	Mid_cent	Gun	3
19	L	Red	Btm_rt	Ball	6	14	yrc(C_OS)	Red	Btm_cent	Ball	6
20	C	Yellow	Mid_rt	M’kb’tle	3	4	CLO_	Yellow	Mid_rt	M’kb’tle	4
21	S	Grey	Top_rt	Sc’driver	2	2	CLOS	Grey	Top_rt	Sc’driver	2
22	O	Orange	Mid_lft	Comb	3	5	CLO_	Orange	Mid_lft	Comb	5
23	L	Blue	Btm_lft	Cup	7	7	ygs(__OS)	Green	Top_lft	Cup	7
24	S	Blue	Btm_lft	Cup	7	23	Ans0	Blue	Btm_lft	Cup	7
25	O	Brown	Mid_lft	Fork	2	10	yrc(CL__)	Brown	Mid_lft	Comb	7
26	C	Pink	Mid_lft	Scissors	3	22	yrc(_L_S)	Orange	Mid_lft	Comb	3
27	L	Yellow	Mid_cent	M’kb’tle	3	20	yrc(C_OS)	Yellow	Mid_rt	M’kb’tle	3
28	O	Pink	Top_rt	Flute	2	21	yrc(_L_S)	Grey	Top_rt	Sc’driver	2
29	S	Yellow	Mid_cent	M’kb’tle	4	11	Ans	Yellow	Mid_cent	M’kb’tle	4
30	C	Grey	Top_rt	Fork	2	2	CL_S	Grey	Top_rt	Sc’driver	2
31	L	Orange	Mid_lft	Comb	5	5	CLOS	Orange	Mid_lft	Comb	5
32	C	Red	Btm_lft	Ball	5	13	crc(C_OS)	Red	Btm_cent	Ball	5
33	O	Blue	Btm_cent	Cup	6	24	crc(C_O_)	Blue	Btm_lft	Cup	7
34	S	Red	Btm_lft	Ball	6	8	ygs(CLO_)	Red	Btm_lft	Ball	8
35	L	Brown	Top_cent	Fork	1	25	yrc(C_O_)	Brown	Mid_lft	Fork	2
36	C	Blue	Btm_lft	Ball	6	34	yrc(_LOS)	Red	Btm_lft	Ball	6
37	S	Orange	Top_rt	Gun	9	12	yrc(_LO_)	Brown	Top_rt	Gun	3
38	L	Grey	Mid_rt	Fork	2	30	yrc(C_OS)	Grey	Top_rt	Fork	2
39	O	Brown	Top_rt	Sc’driver	1	35	yrc(C__S)	Brown	Top_cent	Fork	1
40	C	Orange	Mid_lft	Comb	4	5	CLO_	Orange	Mid_lft	Comb	5
41	S	Green	Mid_lft	Scissors	8	26	yrc(_LO_)	Pink	Mid_lft	Scissors	3
42	C	Green	Mid_lft	Scissors	3	26	yrc(_LOS)	Pink	Mid_lft	Scissors	3
43	L	Blue	Btm_cent	Ball	4	36	yrc(C_O_)	Blue	Btm_lft	Ball	6
44	S	Black	Top_rt	Gun	1	39	crc(_L_S)	Brown	Top_rt	Sc’driver	1
45	O	Black	Top_rt	Gun	1	44	Ans0	Black	Top_rt	Gun	1

Columns 3–6 give the combination of cue and responses actually recorded; Columns 9–12 reproduce the presumed source of the responses. Column 7 is the trial number of the source (less than 10 means the source is a stimulus) and Column 8 the nature of the match (CLOS = completely correct; CLO_, CL_S, C_OS, _LOS = two responses correct [using ‘_’ to indicate an incorrect response]; CL__ etc. = one response correct, Ans = complete recall of a previous erroneous trial; crc = cued recall of 1 or 2 responses; yrc = 2 or 3 responses, none of them matching the cue; ygs = 2 or 3 responses matching a wrong stimulus). Null signifies three independent guesses with no identifiable source

There are also different patterns of repetition to be distinguished. The combination of cue and three responses on a trial will be called an *answer*. The individual components of an answer are the *cue* and (three) *responses*. Where one answer exactly matches the cue and three responses on some previous trial (but not a correct recall of a stimulus, Trial 29), I speak of the recall of an *answer*; but it will be necessary to distinguish answers that are copies of the answer on the immediately preceding trial, *answer (lag 0)* or, simply, *Ans0* (Trials 24 and 45), because the proportion of *immediate* repetitions is near 1. The answer that is repeated might itself be the recall of a previous error (Trials 35 and 41) or might be an incomplete recall of a stimulus (Trial 11). If only one or two responses (and the cue) match the answer on some previous trial, that is a *cued recall* (crc, Trials 14, 32, 33, 44). In the case that two or three responses, but *not* the cue, match some previous answer, I speak of a *yoked recall* (yrc, many trials in Table 1), and a *yoked guess* is two or three responses that match some stimulus other than the correct one (ygs, Trials 10, 12, 13, 23, 34). Finally, *Null* (Trial 15) signifies three independent guesses.

Given such a frequency and variety of repetition, the next question must be statistical significance. Since, when recall fails, there are 729 different combinations of three attribute values that might comprise a guess, the probability of matching any preceding trial by chance is tiny. The manner of calculating those probabilities is explained later under “Results”, but, anticipating those calculations, the statistical evaluation of different categories of repeated errors in the present experiment is set out in Table 2, where the numbers of answers actually recalled are compared with the calculated probabilities. That comparison between numbers of repetitions and chance probabilities provides the justification for this review. Most of those repeated errors must have been retrieved from memory. Analysis of those repetitions provides a novel means of observing the storage and retrieval of memories in the short term.

**Table 2:** Incidences of different categories of repeated errors in the experiment by Jones (1978), with a statistical assessment of each category

Cue attribute	Response attribute(s)	Total trials	Number observed	95% confidence interval	Proportion	Chance expectation	Chi Square	Significance
*Aggregate lag 0 answer fragments*								
C	LOS	72	51	^a,^0.716	0.708	0.099	162.077^b^	< 0.001
L	COS	67	45	^a,^0.683	0.672	0.092	148.234^b^	< 0.001
O	CLS	65	44	^a,^0.674	0.677	0.089	147.156^b^	< 0.001
S	CLO	75	45	^a,^0.732	0.600	0.103	140.071^b^	< 0.001
*Aggregate lag > 0 answer fragments*					0.276			
C	LOS	508	136	^a,^3.522	0.268	1.2953	14.056.75	< 0.001
L	COS	497	124	^a,^3.352	0.249	1.2047	12.556.89	< 0.001
O	CLS	536	161	^a,^3.568	0.300	1.3201	19.376.47	< 0.001
S	CLO	475	136	^a,^3.476	0.286	1.2706	14.338.48	< 0.001
*Aggregate cued triples*					0.368			
C	LO	289	88	8.564, 23.739	0.304	16.1512	790.5958	< 0.001
	LS		4					
	OS		33					
L	CO	309	84	8.958, 24.410	0.272	16.6837	647.6132	< 0.001
	CS		6					
	OS		27					
O	CL	336	84	9.990, 26.074	0.250	18.032	869.1192	< 0.001
	CS		33					
	LS		22					
S	CL	274	12	8.608, 23.736	0.044	16.1718	147.224	< 0.001
	CO		33					
	LO		18					
*Aggregate yoked triples*					0.105			
C	LOS	487	48	3.064, 14.569	0.099	8.8164	178.25	< 0.001
L	COS	526	61	3.306, 15.050	0.116	9.178	299.2099	< 0.001
O	CLS	469	12	2.824, 14.093	0.026	8.4587	1.5174	0.218
S	CLO	558	94	2.881, 14.234	0.168	8.5573	870.4084	< 0.001
*Aggregate three-yoked guesses*					0.103			
C	LOS	332	18	^a,^6.579	0.054	3.1291	71.3689	< 0.001
L	COS	366	26	^a,^7.071	0.071	3.4489	148.9128	< 0.001
O	CLS	334	8	^a,^6.782	0.024	3.2609	6.957	0.008
S	CLO	408	96	^a,^6.708	0.235	3.2113	2704.634	< 0.001
*Aggregate cued pairs*					0.413			
C	L, O, S	128	52	48.299, 68.487	0.406	58.3931	1.5411	0.214
L	C, O, S	148	49	59.253, 80.723	0.331	69.9879	14.6838	< 0.001
O	C, L, S	162	92	67.910, 90.492	0.568	79.201	4.9359	0.026
S	C, L, O	138	45	57.797, 78.531	0.326	68.1639	19.1776	< 0.001
*Aggregate yoked pairs*					0.366			
C	LO	229	30	65.369, 91.609	0.131	78.4891	0.4497	0.502
	LS		28					
	OS		16					
L	CO	243	52	70.584, 97.937	0.214	84.2604	8.0023	0.005
	CS		23					
	OS		29					
O	CL	226	13	65.970, 92.771	0.058	79.3708	8.8771	0.003
	CS		16					
	LS		30					
S	CL	241	16	67.781, 94.797	0.066	81.2888	13.9168	< 0.001
	CO		55					
	LO		36					
*Aggregate two-yoked guesses*					0.513			
C	LO, LS, OS	113	56	^a,^7.414	0.496	3.706	764.0262	< 0.001
L	CO, CS, OS	103	49	^a,^6.993	0.476	3.4272	627.6495	< 0.001
O	CL, CS, LS	84	41	^a,^6.073	0.488	2.8321	532.8968	< 0.001
S	CL, CO, LO	94	56	^a,^5.971	0.596	2.7644	1058.736	< 0.001

^a^The conventional approximation to the lower limit of the 95% confidence interval fails when the chance expectation is very small

^b^Normal deviate from binomial test with probability 1/728

## The experiments

In this article, I examine/re-examine the data from four experiments of which the first, Jones (1978), has already been described.

*Lansdale and Laming* (1995): This study repeated Jones’ (1978) experiment using colour slides of a billiards table with a coloured ball (C: black, blue, brown, cyan, green, orange, pink, white, yellow, but not red) somewhere between the centre pockets, a distinctive pattern (P: nine different patterns) of eight red balls somewhere on the further half of the table and a white object (O: beer mug, book, brush, clock, cup, gloves, milk bottle, newspaper, vase) on the left hand edge of the table. As before, these components were independently assembled to produce nine different presentation sets of nine stimuli such that each component appeared exactly once in each set. Participants viewed the stimuli in a set for 2.1 s each; then, after an interval of 25 s counting backwards by threes, there followed 27 test trials. On each trial participants were cued with one of the components from one of the stimuli and asked to recall the other two. They were asked to guess if they could not recall. Each stimulus was tested at intervals of nine trials in the sequence of 27. Successive trials always presented a different attribute (C, P, O) as cue.

Each participant was asked to study and recall three sets of stimuli in a session of one hour. Twenty-seven participants completed a design balanced across all nine presentation sets. Two very similar experiments were reported. In the first, participants recorded their responses on printed sheets of paper and added their confidence (ratings 1–5) that each response was correct. In the second, the stimuli were presented on a computer peripheral and latencies were measured. Taken together, these two experiments comprised a total of 4374 test trials.

*Laming* (2021): The stimuli were 90 advertisements culled from glossy magazines. Each advert consisted of a brand name (B), a picture (P) of the product and a slogan (S). They were divided into nine product groups (cosmetics, fashion, food and drink, furniture, technology, holidays, jewellery, perfumes and shoes) with ten advertisements in each group. The slides were presented at 15 s intervals using a Kodak carousel projector. Each product group was tested in a separate session, lest the mixing of, say, perfumes and food provided additional cues which brand name, picture and slogan went together. The test trials comprised a series of 30 cues on slides, each slide showing one component from one of the stimuli. A picture cue showed the picture from the original advertisement with the brand and slogan blanked out. Brand names and slogans were typed in Times 12pt, the brand names in capitals, and photographed against a black ground.

There were 30 participants, who were tested on the different product groups at various intervals after presentation, ranging from an immediate test up to 4 months (but the loss of recall with lapse of time is not reviewed here). The cues were ordered such that each stimulus was tested every ten trials and successive cues presented different attributes. Participants were asked to write their responses in booklets^1^ and, to assist, a complete set of brand names, slogans and pictures was projected on a screen. The pictures were labelled A, ⋯, J and the brand names and slogans were ordered alphabetically to obscure any association between them. Participants were instructed to select a guess from this screen if they could not remember; 30 s was allowed for responding to each cue. It is inevitable that over the space of 4 months not all participants will attend all the testing sessions; this experiment yielded a total of 5880 test trials.

*Ross and Bower* (1981, *Expt. 3*)^2^: “The learning materials were groups of four words [quartets] that have slight pre-experimental connections to a common concept, frame, or script (see Schank & Abelson, 1977).” Ross and Bower (p. 5). There were four lists of 12 quartets. The quartets were projected, one at a time, for 10 s onto a screen by an overhead projector. Thereafter, each quartet was cued twice with different component words. Participants were asked to write their recalls of the other three words of the quartet in a booklet, with precautions to prevent them looking back at previous responses. There were 18 participants who studied and recalled four lists in random order, giving a total of 1728 test trials.

These four experiments were originally designed to test the fragmentation hypothesis (Jones, 1976), which says, simply, that the representation of a stimulus in memory fragments. The contents of the fragment (e.g. Colour, Location, Object, Serial position) determine what attributes will function as cue and what other attributes can be retrieved. The most stringent test of this hypothesis subsists in cueing each stimulus by each of its individual components in turn and then examining the consistency of the retrievals. To my knowledge, only these four experiments have tested the hypothesis in this particular manner. Other experiments have also addressed the fragmentation hypothesis, most recently Joensen et al. (2019), but do not identify the sources of responses with the precision achieved here. So, these four experiments are selected for reanalysis because of the data they provide about the sources of individual responses. The fragmentation hypothesis has no further role to play, except that the repetition of previous answers generates data that suggests the fragmentation hypothesis without that hypothesis having any relevance to memory (Laming, 2019).

The data are reanalysed here solely to identify the most probable source for each combination of responses. Such a re-analysis of Lansdale and Laming (1995) has already been published (Laming, 2019) and the initial publication of the third experiment (Laming, 2020) already focuses on repeated errors. (The statistical analyses from these two experiments are placed in a supplementary file (Review_tables.doc) and the results merely summarised below). But the experiments by Jones (1978) and Ross and Bower (1981, Expt. 3) are here re-analysed ab initio, working from the original data. The analysis is set out under ‘Results’; then ‘Three Propositions’ presents some immediate post hoc interpretations and their relation to previous work; further comment is in the Discussion. This arrangement of the argument means that certain topics recur and some sub-headings are repeated three times.

## Results

The reanalysis works through each sequence of test trials in inverse order. Beginning at the end, it seeks the most probable source for each combination of cue and responses. A test of the fragmentation hypothesis would look only at correlations between the responses to the three cues addressing each individual stimulus; the analysis/re-analysis here examines the correlations between each combination of cue and responses and all preceding such combinations, including the original stimuli. The analyses are exploratory and the interpretations below post hoc. The results are subdivided into.

1. repetitions of previous errors;
2. conditional proportions of repetitions; and
3. the relation between lag and recall.

### Repetitions of previous errors

For each trial *n* (*n* = 10, ⋯ 45), the reanalysis works backwards from trial *n* − 1 to 10 and then through the stimulus set (*n* = 9, ⋯ 1), looking for the best match to the cue and three responses. A previous answer (or stimulus) that matches all four attributes is always deemed a more probable source than a match of only three or two. Given two matches of equal size, the most recent (with the smallest lag) is preferred over the more remote.

Suppose the answer on trial *n* is not correct, but matches the cue and three responses on some previous (erroneous) trial. The probability of a complete match by chance, not this particular match, but any match, depends on the number of previous answers that could be matched by a suitable choice of responses. There is some number of previous answers containing the trial cue as a response. Let that number be *x*; it varies from trial to trial, but is usually 0, 1 or 2. There is also some number of combinations of three responses (728, because the stimulus addressed must be excluded from the calculation) that might have been output. The probability of matching a previous answer (any previous answer) by chance is therefore *x*/728. The occurrence of a match is a Bernoulli variable with variance (1 − *x*/728)(*x*/728). The sum of such variables over the totality of trials is a generalised binomial, with mean equal to the sum of the probabilities and variance to the sum of the individual variances. This mean is compared in Table 2 with the numbers of answers actually recalled.

If the output on trial *n* is not a complete repetition of any previous answer or stimulus, it may combine three or two matching attributes. Such a combination may consist of the cue and one or two matching attributes (*a cued pair or triple*) or of two or three attributes excluding the cue (*a yoked pair or triple*). Answers containing one or two correct responses must now be excluded from the calculation to preclude the number of matches being inflated by partial recalls of stimuli. The probabilities of cued pairs and triples and of yoked pairs and triples are calculated, along the lines set out above for an answer, except that additional combinations of cue and responses must now be excluded from the calculation. If the cue is a Colour, there is one combination of Location and Object that would give a triple match to a stimulus; that combination must be excluded. In addition, there may be Serial position values that, when added to one of the other 80 combinations (of Location and Object), would give a complete match to a previous answer. Such a triple combination of guesses is also excluded.

Yoked repetitions are simpler. Three Location, Object and Serial position guesses (excluding the cue) may be matched to any previous answer that does not include the trial cue, else it would be a repetition of a complete answer. It may also be matched to an incorrect stimulus, where it would be separately classified as a *yoked guess*. The Bernoulli probabilities and variances are again summed to provide a generalised binomial variable for comparison with the total number of cued/yoked pairs/triples recalled. These calculations for an experiment with three-attribute stimuli are explained in great detail in Lansdale and Laming (1995, pp. 44–50).

*Jones* (1978): In Table 2 ‘Total trials’ in Col. 3 is the number of trials, conditional on the cue, on which a repetition of the designated category might have been observed. These numbers relate to a total of 972 trials with each attribute as cue, from which quadruple-correct answers (and, for triple-matches, triple-correct and repeated answers) have been deleted. The sums of the Bernoulli probabilities are small in relation to the numbers of previous errors that are repeated, so that most such repetitions must have been true recalls from memory. One might summarise the results as follows: yoked triples and guesses to Object as cue, and several different pair repetitions are not significant (well, not at 0.001).

*Lansdale and Laming* (1995): The statistical evaluation of this experiment has already been published (Laming, 2019) and is here placed in a supplementary file (Table A). There was a total of 1458 trials with each attribute as cue. The repetition of complete answers is again highly significant. Of pair repetitions, C and O pair together, both as cued and as yoked recalls, but pair combinations involving P are generally not significant. Colour and Object, of course, have natural names; Pattern does not.

*Laming* (2021): The statistical evaluation of this experiment is also placed in a supplementary file (Table B). There was a total of 1960 trials with each attribute as cue. Excepting yoked guesses, everything is significant at 0.001, except for pair-repetitions that do not include the picture. Briefly, the brand might be recalled with the picture, or the slogan, but without the picture, brand and slogan are lost. The picture plays a pivotal role in the recall of these advertisements.

*Ross and Bower* (1981,* Expt. 3*): As with Jones (1978) and the other two experiments, each set of responses was presumed to be a recall of the previous trial or stimulus with which it had the greatest congruence. Where two sources showed equal congruence, the most recent was preferred, so that the second complete recall of a quartet was sourced to the first recall. Where recall was incomplete, participants would sometimes produce additional words from other quartets, quartets other than the correct one. Such additional words are treated as true recalls from a previous trial or stimulus, choosing the most recent when there was more than one such source. Intrusions from previous lists or from outside the experiment altogether are ignored.

The participants were not instructed to guess if they failed to recall and certainly did not have a finite pool of words from which the probability of a repeated recall could be calculated. It is not therefore possible to assess the statistical significance of the aggregate repetitions. Instead, Table 3 below simply records the numbers of each category of recall. The categories are ‘single’, ‘double’ and ‘triple retrieval’, and if ‘Cue’ is included, the recall is obtained either from a stimulus or from a previous recall of a stimulus (i.e. a correct recall). If there is no mention of ‘Cue’, the responses have been retrieved from some other preceding trial (i.e. an error). ‘Cue only’ signifies no recall at all. Table 3 further separates retrievals according as they are sourced to a stimulus, to a previous trial, or to a previous trial that was itself a recall of a previous trial.

**Table 3:** Numbers of different categories of retrieval in Ross and Bower (1981, Expt 3)

Source	Cue only	Category of recall					
		Single retrieval	Cue and single retrieval	Double retrieval	Cue and double retrieval	Triple retrieval	Complete recall
Stimulus	727	75	201	7	156	3	267
Previous recall	15	53	41	17	74	4	236
Recall of a recall	1	18	2	3	5	1	3

*Summing up: repetitions of previous errors*: In the data from Jones (1978), Lansdale and Laming (1995) and Laming (2021) many previous errors are repeated in recall. Individual categories of repetition are typically significant at 0.001. The exceptions are triples and pairs that lack a particular component (Object in Jones, 1978, Picture in Laming, 2020) or include Pattern in Lansdale and Laming (1995). The experiment by Ross and Bower (1981) shows a much greater proportion of completely correct recalls and the prevalence of repeated errors is accordingly less.

### Conditional proportions of repetitions

**Fig. 2:**
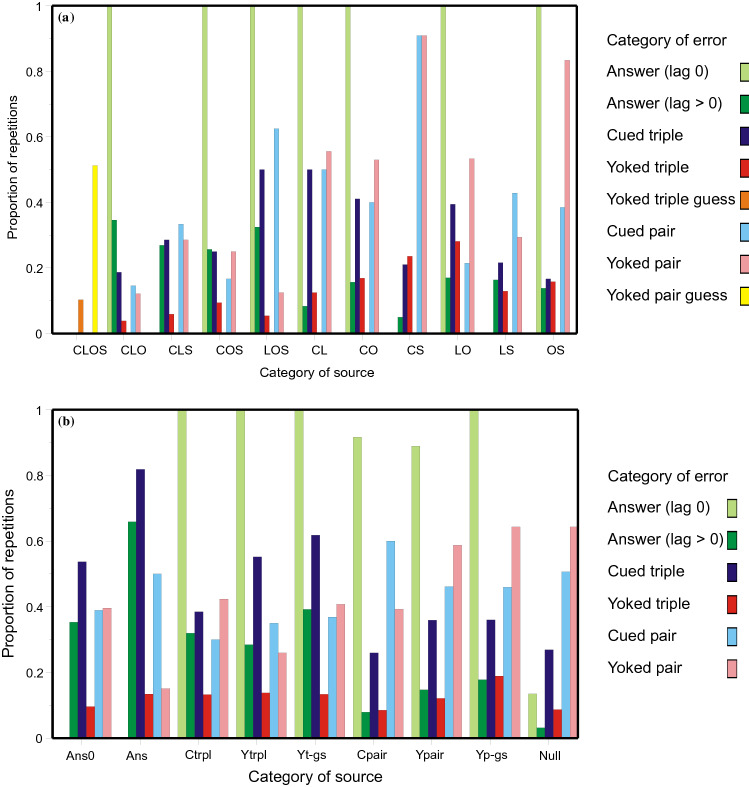
Proportions of errors repeated conditional on the nature of the source: **a** correct or partially correct sources; **b** errors. Each cluster of histogram bars records the proportions of errors conditional on the source below. The different sources are CLOS, CLO, CLS, COS, LOS, CL, CO, CS, LO, LS, OS, Answer (lag 0), Answer (lag > 0), Cued triple, Yoked triple, Yoked triple guess, Cued pair, Yoked pair, Yoked pair guess, and Null. Data from Jones (1978)

Many errors are retrievals of previous errors; Figs. 2, 4 and 6 display conditional proportions of retrievals, conditional on the source. For example, the pale green histogram bars are where the cue on trial *n* + 1 happens to be one of the responses made on trial *n*. These bars show the proportions, conditional on the category of error on trial *n*, of a complete reproduction of the preceding set of responses (*Ans0*). The proportions are all near 1, except when trial *n* is Null, composed of two or three independent guesses. Likewise, the dark green histogram bars show the corresponding proportions for answers with lag > 0. The proportion conditional on a previous (identical) answer as source is greater than the rest. Putting this more generally, the entries in Table 2 (and Tables A an B in Review_tables.doc), already classified according to category of error; can be further decomposed according to the category of the presumed source. Each entry under ‘Number observed’ (col. 4) has already been traced to a source. For each trial enumerated under ‘Total trials’ (col. 3) there are one or more prior trials that could have been retrieved to generate that particular category of error, and I take the most recent of those prior trials as the potential source. The proportions in Figs. 2, 4 and 6 are the quotients of these two quantities.

The repetition of (erroneous) answers applies equally to correct recalls. A completely correct recall on first cueing places an additional copy of the stimulus in memory, which supports an increased likelihood of a correct recall at the next test, while a failure to recall has the contrary effect. There is no feedback following any of these tests, no further sight of the stimulus, so that the sequence of successive recalls should be a martingale (see Laming, 2005).^3^ While the probabilities following particular sequences of recalls diverge, the expectation remains unchanged.

**Fig. 3:**
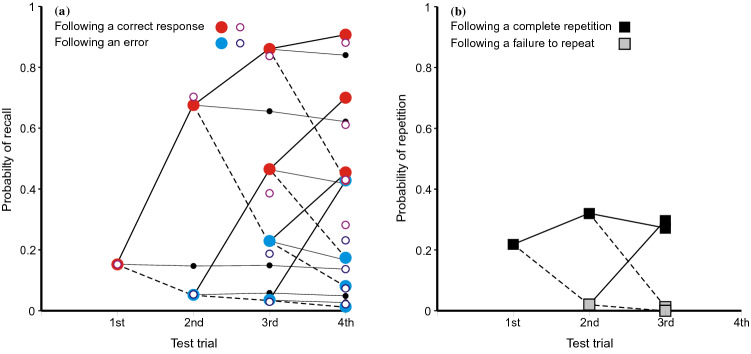
**a** Proportions of completely correct (CLOS) answers as a function of ordinal number of trial and of outcomes on preceding trials from Jones (1978). The small black circles represent the mean proportions correct on the second and third and fourth trials; they are linked to the preceding data points to emphasise that the process is a martingale. The open circles are predictions from the model in the "Appendix". **b** The corresponding proportions for repeated answers. Data from Jones (1978).

*Jones* (1978): Figure 2 decomposes the entries in Table 2 according to the category of the presumed source. Different categories of source are represented by different clusters of histogram bars spaced along the abscissa. Within each cluster different coloured bars show the quotient of the number of repetitions in that category (of repetition) divided by the number of trials on which such a repetition might have been observed.

There are two significant features. First, if the trial cue on trial *n* + 1 is also one of the responses on the immediately preceding trial (*n*), repetition of that preceding trial (*Ans0*) is almost certain, except in the case that the preceding trial is Null (three unrelated guesses). The average proportion of repetitions (Null sources excepted) is 0.985; it does not vary significantly between sources [*χ*^2^(*N* = 175) = 10.653, with 7 *df*, *p* = 0.154], while retrievals from Null sources (0.135) are much less frequent [*χ*^2^(*N* = 279) = 105.620, with 1 *df*, *p* < 0.001]. Second, the average proportion of repetitions for answers (lag > 0) is 0.232, but increases to 0.658 when the source is itself an (identical) answer. Likewise, the average proportion of repetitions for a cued triple is 0.383, but increases to 0.818 when the source is an answer. Complete (erroneous) answers tend especially to be repeated.

Figure 3 shows the proportions of completely correct (CLOS) answers following each sequence of preceding correct and incorrect trial responses for that particular stimulus. The proportion correct on first test is 0.152 and, if subsequent tests depended solely on independent access to the stimulus, the proportion of four correct CLOS answers would be 0.0005. In fact, the proportion of four correct CLOS answers in sequence is 0.080. It is plain from Fig. 3 that a correct (CLOS) answer increases the conditional probability of a correct answer on the next test. The small black dots mark the means at the second, third and fourth trials. They are linked by thin black lines to the corresponding data points representing the proportions correct on the preceding trial. This is to emphasise that while the probabilities for individual sequences diverge, the expectations do not.

The open circles in Fig. 3a are calculations from a model in the "Appendix".^4^ There are four trials for each stimulus spaced at intervals of nine within the trial sequence; the combinations of responses on those trials have different accessibilities (probabilities of retrieval). The probability of retrieving the original stimulus, *a*_s,_ is assumed constant, but thereafter *a*_1_ is the accessibility of the responses on the previous trial (the first trial at the second test, but the second trial at the third test and the third trial at the fourth test). At the second and third tests, *a*_2_ is the accessibility of the preceding test but one, while *a*_3_ is the accessibility of the first test at the fourth trial. The parameter estimates are: *â*_s_ = 0.152 for the stimulus, *â*_1_ = 0.650 for the preceding trial, *â*_2_ = 0.451 and *a*_3_ = 0.270. There are 16 terminal proportions to be modelled with four free parameters [*χ*^2^(*N* = 972) = 13.417, with 11 *df*, *p* = 0.267].

The black squares in Fig. 3b show the corresponding proportions for repeated answers. At first test they show the proportion of answers (lag > 0) sourced from triple, pair and Null sources (0.218) and, at subsequent tests, the proportion sourced from previous answers (0.320, 0.271).

*Lansdale and Laming* (1995): Figure 4 decomposes the entries in Table A in Review_tables.doc in like manner to Fig. 2. As before, if the trial cue matches one of the responses on the immediately preceding trial, repetition of that preceding answer (lag 0) is almost certain, except in the case that the preceding trial is Null (two unrelated guesses). The average proportion of repetitions (Null sources excepted) is 0.971; it does not vary significantly between sources [*χ*^2^(*N* = 173) = 10.922, with 7 *df*, *p* = 0.142], while retrievals from Null sources (0.328) are much less frequent [*χ*^2^(*N* = 432) = 213.013, with 1 *df*, *p* < 0.001]. The average proportion of repetitions for answers (lag > 0) is 0.158, but increases to 0.514 (see Fig. 5 below) when the source is itself an (identical) answer.

**Fig. 4:**
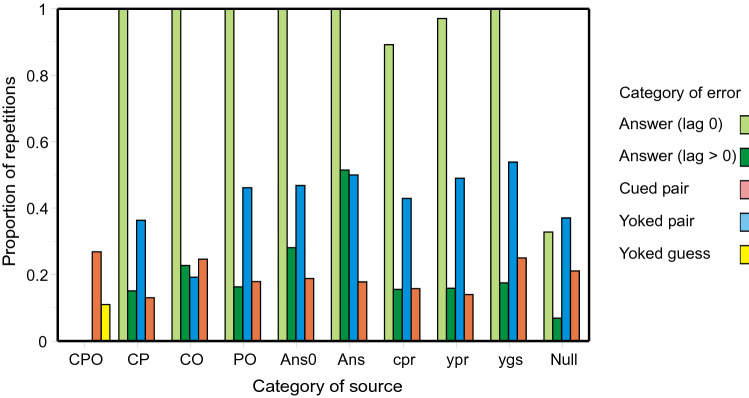
Proportions of errors repeated conditional on the nature of the source. Each cluster of histogram bars records the proportions of errors conditional on the source below. The different sources are CPO, CP, CO, PO, Answer (lag 0), Answer (lag > 0), Cued pair, Yoked pair, Yoked pair guess, and Null. Data from Lansdale and Laming (1995)

**Fig. 5:**
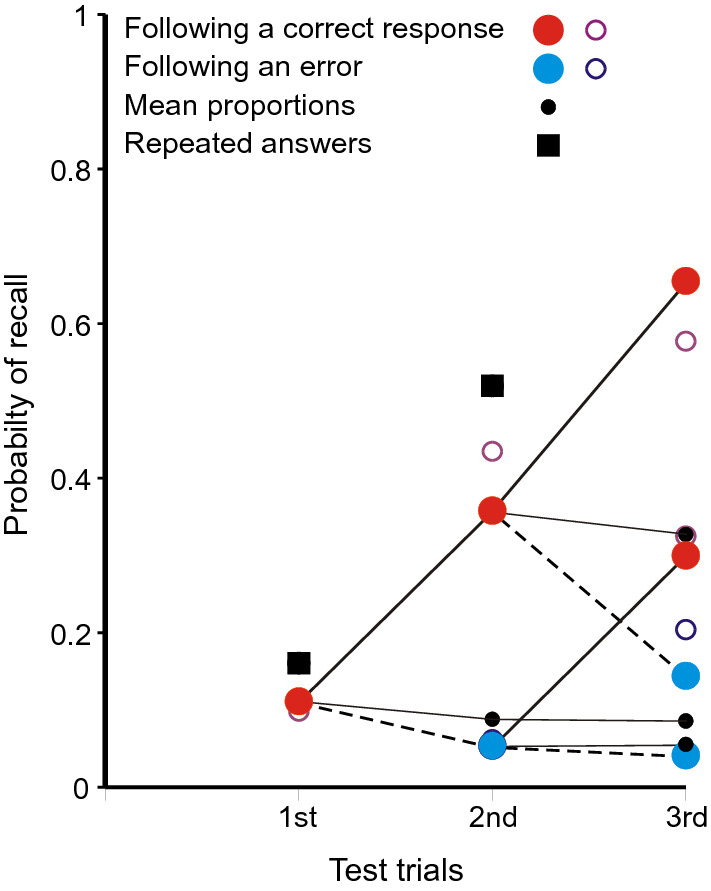
Proportions of completely correct (CPO) answers as a function of ordinal number of trial and outcomes on preceding tests from Lansdale and Laming (1995). The corresponding proportions for repeated answers are shown for comparison. The small black circles represent the mean proportions correct on the second and third trials; they are linked to the preceding data points to emphasise that the process is a martingale. The open circles are predictions from the model in the "Appendix". Data from Lansdale and Laming (1995)

Figure 5 shows the proportions of completely correct (CPO) answers following each sequence of preceding correct and incorrect trials. The proportion correct on first test is 0.111 and, if subsequent tests depended solely on independent access to the stimulus, the proportion of three correct CPO answers to a stimulus would be 0.0014. In fact, the proportion of three correct CPO answers in sequence is 0.0261. As before, a correct (CPO) answer increases the conditional probability of a correct answer on the next test. The small black circles show the means at the second and third trials, linked by thin black lines to the data points representing the proportions correct on the preceding trial. The open circles are predictions from the model in the "Appendix". The parameter estimates (cf. above) are: *â*_s_ = 0.098 for the stimulus, *â*_1_ = 0.373 for the preceding trial and *â*_2_ = 0.252 [*χ*^2^(*N* = 1458) = 12.658, with 4 *df*, *p* = 0.013].

The black squares in Fig. 5 show the corresponding proportions for repeated answers. At first test they show the proportion of answers (lag > 0) sourced from pair and Null sources (0.158) and, at second test, the proportion of answers sourced from existing answers (0.514). In both cases the proportions of answers retrieved exceed those of correct recalls [1st test: *χ*^2^(*N* = 3534) = 9.246, with 1 *df*, *p* = 0.002; 2nd test: *χ*^2^(*N* = 266) = 6.738, with 1 *df*, *p* = 0.009].

**Fig. 6:**
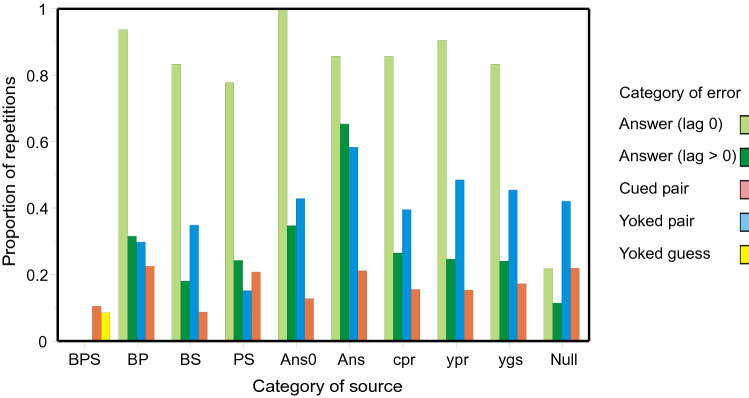
Proportions of errors repeated conditional on the nature of the source. Each cluster of histogram bars records the proportions of errors conditional on the source below. The different sources are BPS, BP, BS, PS, Answer (lag 0), Answer (lag > 0), Cued pair, Yoked pair, Yoked pair guess, and Null. Data from from Laming, (2021)

*Laming* (2021): Figure 6 decomposes the entries from the recall of advertisements in Table B in Review_tables.doc in like manner to Fig. 2. As above, if the trial cue matches one of the responses on the immediately preceding trial, repetition of that preceding answer (lag 0) is highly probable, except in the case that the preceding trial is Null. The average proportion of repetitions (Null sources excepted) is 0.835; it varies somewhat between sources [*χ*^2^(*N* = 121) = 18.403, with 8 *df*, *p* = 0.018], while retrievals from Null sources (0.218) are much less frequent [*χ*^2^(*N* = 286) = 114.362, with 1 *df*, *p* < 0.001]. Second, the average proportion of repetitions for answers (lag > 0) is 0.244, but increases to 0.653 (see Fig. 6 below) when the source is itself an (identical) answer.

**Fig. 7:**
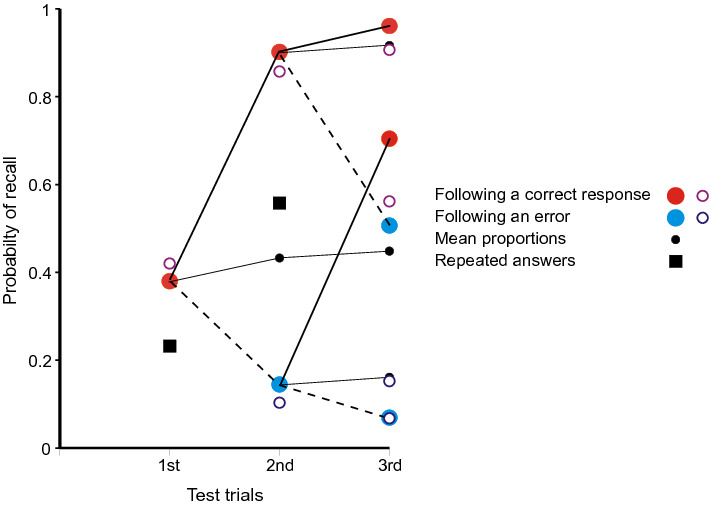
Proportions of completely correct (BPS) answers as a function of ordinal number of trial and outcomes on preceding tests from Laming, (2021). The corresponding proportions for repeated answers are shown for comparison. The small black circles represent the mean proportions correct on the second and third trials; they are linked to the preceding data points to emphasise that the process is a martingale. The open circles are predictions from the model the "Appendix". Data from Laming, (2021)

Figure 7 shows the proportions of completely correct (BPS) answers following each sequence of preceding correct and incorrect trials addressing that stimulus. The proportion correct on first test is 0.380 and, if subsequent tests depended solely on independent access to the stimulus, the proportion of three correct BPS answers would be 0.055. In fact, the proportion of three correct BPS answers in sequence is 0.330. A correct (BPS) answer greatly increases the conditional probability of a correct answer on the next test. As above, the small black circles show the means at the second and third trials, linked by thin black lines to the data points representing the proportions correct on the preceding trial. The open circles in Fig. 7 show predictions from the model in the "Appendix". The parameter estimates are: *â*_s_ = 0.420 for the stimulus, *â*_1_ = 0.754 for the preceding trial and *â*_2_ = 0.345.

The black squares in Fig. 7 show the corresponding proportions for repeated answers. At first test they show the proportion of answers (lag > 0) sourced from pair and Null sources (0.209) and, at second test, the proportion of answers sourced from previous answers (0.653).

**Fig. 8:**
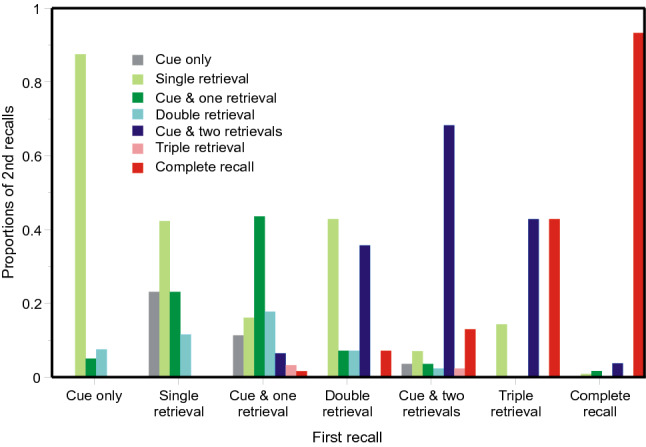
Proportions of categories of recall on second recall for each different category of first recall in the experiment by Ross and Bower (1981). The first recall might comprise a complete recall, or two correct recalls (cue and two retrievals) or a single correct recall (cue and one retrieval). Alternatively, there might be three words from the same wrong source (Triple retrieval) or 2 words (Double retrieval) or just one word (Single retrieval) or nothing (Cue only). Data from Ross and Bower (1981)

*Ross and Bower* (1981, Expt. 3): Notwithstanding that each stimulus (quartet) had four components, the stimuli were cued only twice. The histogram clusters in Fig. 8 present the distributions over different categories of second recall, correct recalls as well as errors, for each different category of first recall. Since there was no set of alternatives from which to select a guess, conditional probabilities cannot be calculated (cp. Figs. 2, 4, 6). If a recall contains the trial cue (Cue and 1, 2 or 3 retrievals), it has been retrieved from the quartet addressed by that cue (or a previous recall of that quartet). The second recall, in this case, tends to duplicate the first. If the recall does not contain the trial cue (1, 2 or 3 retrievals only), it necessarily comes from some other source and is an error.

**Fig. 9:**
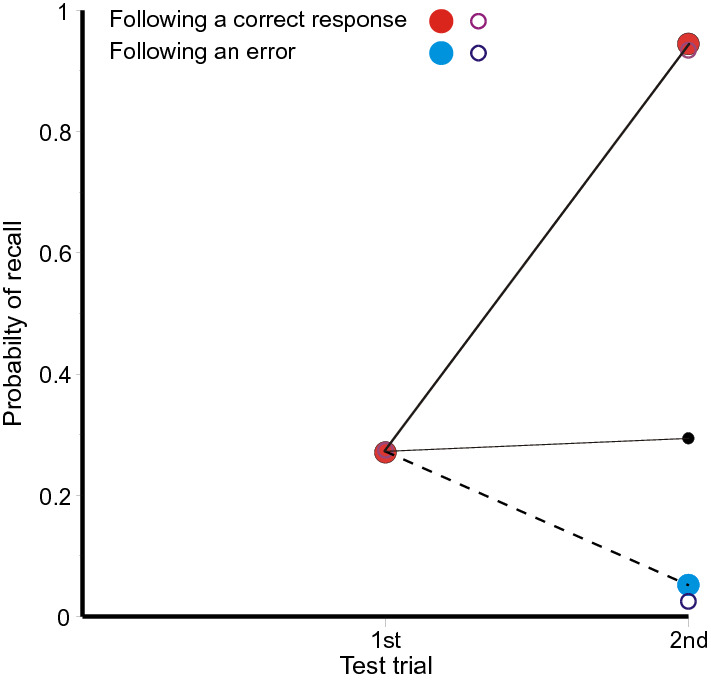
Proportions of completely correct quartets as a function of ordinal number of trial and the outcome on a preceding trial in the experiment by Ross and Bower (1981). The small black circle represents the mean proportion correct on the second trial; it is linked to the preceding data point to emphasise that the process is a martingale. The open circles are predictions from the model in the "Appendix". Data from Ross and Bower (1981)

Figure 9 shows the proportions of completely correct recalls on first and second tests. If the first recall is completely correct, the probability of a completely correct recall on second test is greatly increased, with a complementary decrease if the first recall fails. The small black circle shows the mean proportion correct on second test and is but little increased over the proportion on first test. The process is a martingale. The open circles are predictions from a simplified version of the model in the "Appendix". In this model a recall is completely correct if either the cue accesses the original quartet (*a*_*s*_ = 0.274) or a correct first recall (*a*_1_ = 0.909).

*Summing up: conditional proportions of repetitions*: Repeated errors are often sourced from previous repetitions. This is abundantly clear in the first three experiments where conditional probabilities can be calculated; it is less apparent in Ross and Bower (1981, Expt. 3). The relation between source and repetition is of no apparent significance except for repetitions of the immediately preceding trial and for repeated answers. If a trial cue turns out to be one of the responses on the immediately preceding trial, the probability of a complete repetition is so near to 1 that there is no significant difference between sources, except when the source is Null. A similar, but more muted, relationship is seen in the repetition of complete (erroneous) answers. The same process applies to completely correct recalls, where it generates a martingale. Individual (conditional) sequences diverge from one test to the next, but the unconditional expected proportion of correct recalls does not change. These properties are apparent in all three experiments where conditional probabilities can be calculated. The data from Ross and Bower (1981, Expt. 3) display the martingale property over two successive tests.

### The relation between lag and recall

Each recall of a previous error, complete answer or cued or yoked triple or pair, may be sourced from any preceding trial. Lag is the difference in trial number between the present trial and the presumed source, less 1, so that a repetition of the immediately preceding trial has lag 0. Given two matches of equal congruence, the most recent (with the shortest lag) is preferred over the more remote. This might appear to bias the lag-recall-relation to shorter lags, but this is illusory. What is presented in the figures below is the probability of repetition. While the number of each sort of error retrieved is constrained to the most recent source, so also is the number of opportunities for such a retrieval. The figures below plot the quotient of number of repetitions over number of opportunities, both constrained to the most recent source, and show how that estimated probability varies with lag.

**Fig. 10:**
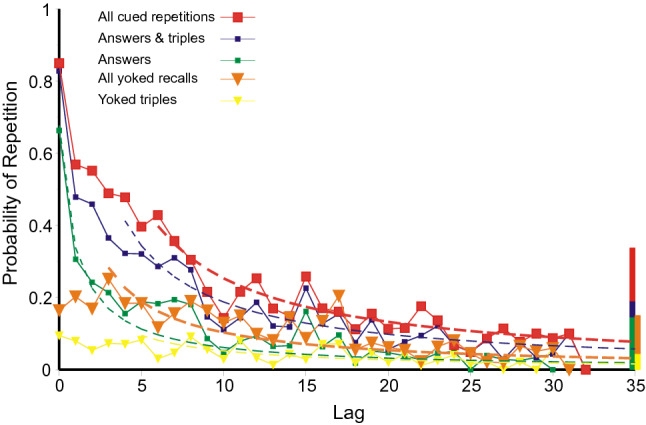
Lag–recall curves for answers, cued triples and pairs and yoked triples and pairs from Jones (1978). The histogram bars at the right hand end of the abscissa are the cumulative proportions of, respectively, complete correct (CLOS) recalls, correct triple recalls and pair recalls and yoked triple and pair guesses. The dashed curves are hyperbolas (Eq. 1). Data from Jones (1978)

**Fig. 11:**
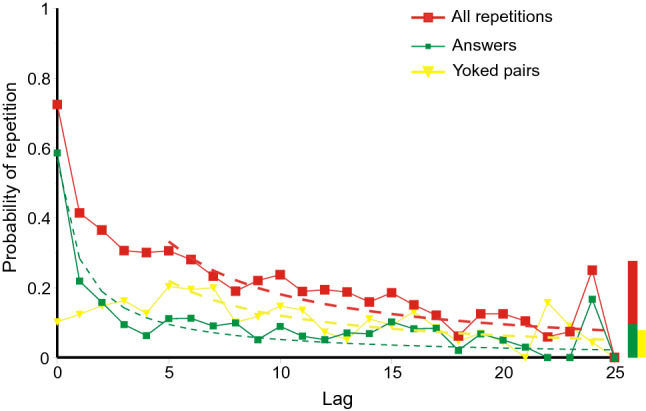
Lag–recall curves for answers, cued pairs and yoked pairs in Lansdale and Laming (1995). The histogram at the right hand end reproduces the cumulative proportions recalled from the stimuli (correct CPO and pair recalls and yoked guesses). The dashed curves are hyperbolas (Eq. 1). Data from Lansdale and Laming (1995)

**Fig. 12:**
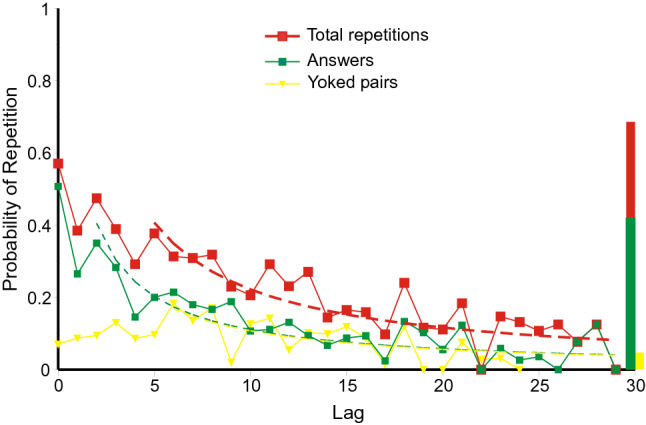
Lag–recall curves for answers, cued pairs and yoked pairs in Laming (2021). The histogram at the right hand side reproduces the cumulative proportions recalled from the stimuli (correct CPO and pair recalls and yoked guesses). The dashed curves are hyperbolas (Eq. 1). Data from Laming (2021)

A cued repetition can originate only from a preceding trial containing the current trial cue, so that answer, cued triple and pair are mutually exclusive. The numbers of answers etc. that might be repeated decreases progressively as lag increases, and the lag-recall relation becomes increasingly variable. Accordingly, these data are plotted cumulatively in Figs. 10, 11, and 12, that is, as ‘Answers’, ‘Answers and triples’, and as ‘All cued repetitions’. The cumulative totals show less variation, trial to trial, and the trend is clearer. For the same reason yoked recalls in Fig. 10 are also cumulated. They are, however, separated from cued repetitions because the essential condition for a yoked recall is that the source does not contain the current trial cue (else the yoked recall would be classified as a answer or cued triple). They originate from those previous trials from which a cued repetition cannot be sourced. There are eight times as many such trials and, to maintain comparability, the proportions of yoked triples and pairs have been increased eightfold.

What is the functional form of the relation between lag and probability of repetition? To provide a basis for comparison, I have chosen to fit the hyperbola.

1 $$ p_{{{\text{lag}}}} \, = \,{\text{A}}_{{{\text{cat}}}} /\left( {{\text{1}}\, + \,r \times {\text{lag}}} \right), $$

where the constant *A*_cat_, different for different categories of repetition, adjusts each curve to the level of repetitions, and the coefficient *r*, fixed for any one experiment, adjusts that relation to the succession of trials. This question is usually asked with respect to time, and here the trial sequence must stand proxy. The constants in Eq. (1) were estimated by least squares, weighted by the number of cases at each lag.

*Jones* (1978): Figure 10 plots the proportions of occasions that the trial at lag *l* is the presumed source for each kind of error, answer or, cued or yoked, triple or pair. Repetitions of each of these kinds of error show conventional recency except that yoked pairs show a depression over the first three lags (≤ 2). (This might not appear noteworthy, but see below). The proportions decrease from about 0.8 for the immediately preceding answer or pair to near zero at a lag of 32. The histogram bars at the right hand end of the abscissa are the cumulative proportions of, respectively, complete correct (CLOS) recalls, correct triple recalls and pair recalls, and triple and pair yoked guesses. The proportions of complete correct (CLOS) recalls, correct triple recalls and pair recalls are comparable to the repetition of answers, cued triples and pairs at about lag 7, while the proportions of triple and pair yoked guesses match the inflated (8 ×) proportions of triple and pair yoked retrievals at about the same lag.

Equation 1 provides a passable representation of the repetition of answers, but not of any other cumulative category unless (as in Fig. 10) the estimated probabilities for the first few lags are excluded.

*Lansdale and Laming* (1995): Figure 11 plots the proportions of occasions that the trial at lag *l* is the presumed source for each kind of error.^5^ The proportions decrease from about 0.5 for the immediately preceding answer or pair to near zero at a lag of 25. As before, the proportions for yoked pairs have been increased eight-fold: Independently of this adjustment, yoked pairs show inverse recency up to lag 7 (Kendall rank correlation 0.714, *p* = 0.007, one-tailed, comparing lags 0–7 only); thereafter the proportions decrease much as do the proportions of answers.^6^ The histogram at the right hand end of the abscissa in Fig. 11 reproduces the cumulative proportions of, respectively, complete correct (CPO) recalls, correct pair recalls and yoked guesses. In each case, the proportions of correct recalls are comparable to the recall of answers, cued pairs and yoked pairs at about lag 5.

Equation 1 provides a passable representation of the repetition of answers, but not of cued or yoked pairs unless (as in Fig. 11) the estimated probabilities for the first five lags are excluded.

*Laming* (2021): Figure 12 plots the proportions of occasions that the trial at lag *l* is the presumed source for each kind of error. The proportions decrease from about 0.5 for the immediately preceding answer or pair to near zero at a lag of 30. As before, the proportions for yoked pairs have been increased, now ninefold, because the stimuli were cued every ten trials. Independently of this adjustment, yoked pairs show inverse recency up to lag 8 (Kendall rank correlation 0.500, *p* = 0.030, one-tailed, comparing lags 0–8 only); thereafter their probabilities decrease much as do those for answers. The histogram at the right hand end of the abscissa in Fig. 12 reproduces the cumulative proportions of, respectively, complete correct (BPS) recalls, correct pair recalls and yoked guesses. In this case the proportions of correct recalls are comparable to the recall of answers and cued pairs at very short lag. Equation 1 provides passable representations of the repetitions only if the first few lags are excluded (see Fig. 12).

**Fig. 13:**
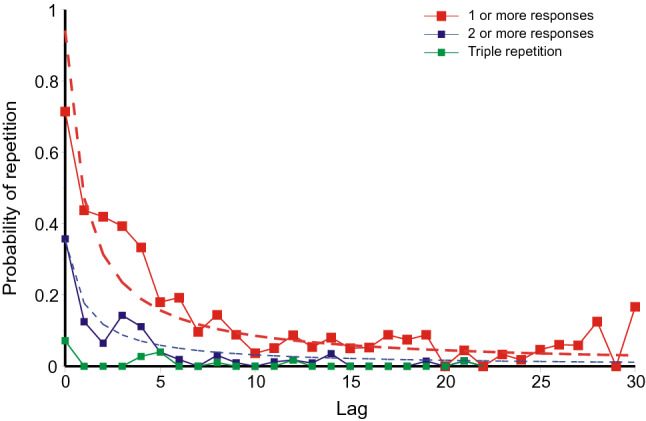
Proportions of repeated errors as a function of lag in the experiment by Ross and Bower (1981). The curves are hyperbolas (Eq. 1, with *r* = 0.669). Data from Ross and Bower (1981)

*Ross and Bower* (1981, *Expt. 3*): This experiment yielded more correct and partially correct recalls than the previous three and correspondingly fewer repeated errors. Figure 13 shows those errors as a function of lag. Each trial was sourced by running backwards in the sequence of trials to find the maximum match, with the trial cue, not itself a response, excluded. The repetitions in Fig. 13 are those for which the maximum match did not contain the trial cue (i.e. errors). Recalls of 1, 2 or 3 words from lag *l* are mutually exclusive, so these entries have been cumulated as in Figs. 10, 11 and 12. Two sets of data points have been fitted with Eq. (1) with the parameters again estimated by weighted least squares. The additional parameter *r* has the value 0.669. It should also be noted that repetitions from lag 0 have high probability.

**Fig. 14:**
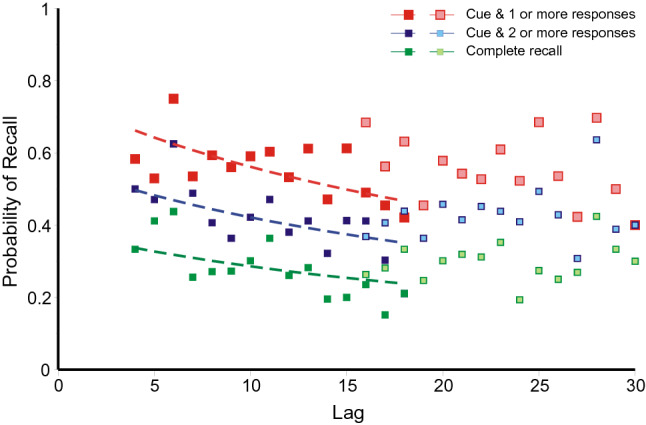
Correct and partially correct recalls as a function of lag in the experiment by Ross and Bower (1981). Each quartet was tested twice; the filled symbols show the results of the first test, the open symbols the second. The curves are hyperbolas (Eq. (1), with *r* = 0.034)

Ross and Bower cued each quartet twice, with different words as cue, at a range of different trial intervals. Figure 14 shows correct recalls, complete or partial, as a function of lag, where lag is trial distance from stimulus – 1. The filled symbols show recalls on the first test, the open symbols recalls on the second. Comparison with Fig. 13 shows the rate of loss of accessibility to be much slower (*r* = 0.034), one twentieth of the former rate; and responses on the second test trial, where there are two entries in memory to access, appear not to show any trend at all.

*Summing up: the relation between lag and recall*: The results from the first three experiments are generally consistent. Figures 10, 11 and 12 present a picture of memory function over the short term; the repetitions equate to a direct observation of individual retrievals. But the record is almost certainly not complete. All the first three experiments show conventional recency (cf. Tan & Ward, 2000) for most categories of repetition, but *inverse recency* for yoked pairs at the lowest lags; this suggests an interaction between different categories of retrieval (see Retrieval As A Function Of Lag/Inverse recency below). The repeated errors in Ross and Bower (1981, see Fig. 13) follow a similar pattern; but correct and partially correct recalls of the original quartets lose accessibility at a much slower rate.

Equation 1 provides a guide to the curvature of the data. But the functional form of the relation between lag and probability of repetition is complicated by the presence of multiple sources. If there have been previous correct recalls of a particular stimulus (Figs. 3, 5, 7, 9), the probability of a further correct recall is much increased. This is true also for repetitions of complete answers. Even so, the repetition of complete answers has arguably the smallest number of alternative sources and presents the clearest picture of the lag-recall relation. In Jones (1978) only 158 out of 557 repetitions of answers were sourced from a previous answer, admitting more than one possible source. In Lansdale and Laming (1995) the proportion was 54 out of 357 and in Laming (2021) 96 out of 495. Amongst simple formulae, Eq. (1) is passable, but many other functional forms could be substituted with suitable values to their constants.

The rate of loss of accessibility is usually estimated with respect to elapsed time; here the succession of trials stands proxy. Equation 1 has a constant *A*_cat_ to adjust to different levels of repetitions, and a coefficient *r* to adjust to the succession of trials. That coefficient is forced to be the same for all categories in an experiment and the estimated values are set out in Table 4 together with the estimated duration of trials. That temporal duration does not differ much between experiments; neither does the rate parameter (*r*) except for correct recalls in the experiment by Ross and Bower (1981), where the loss of accessibility of correct recalls proceeds at only 1/20th of the rate for repeated errors. I emphasise that those two rates refer to responses made by the same participants in the same series of trials. The only difference is the relation of the responses to the quartet addressed by the cue.

**Table 4:** Estimated rate parameters for the hyperbolas in Figs. 10, 11, 12, 13 and 14

Experiment	Rate of succession of trials	*r*
Jones (1978)	Self-paced, c 20 s/trial	0.606
Lansdale and Laming (1995)	Self-paced, c 30 s/trial	1.085
Laming (2021)	30 s/trial	0.347
Ross and Bower (1981, Expt. 3)	Self-paced, c 20 s/trial	
Repeated errors		0.669
Correct recalls, 1st test only		0.034

## Three propositions

The repetition of errors has not previously been studied. This review has assembled the results of four experiments to spell out their implications for our understanding of memory. I set out three propositions concerning what one might call the mechanics of memory. References to previous work will illustrate the wider applicability of these propositions:

1. Every event (a stimulus or a response or just a retrieval) to which the participant attends is separately recorded in memory, creating an ordered record of those events that have engaged the participant’s attention.

### Repetition of errors

Table 5 sets out the proportions of each kind of repeated error in these experiments. In Jones (1978) there was a repeated error of some kind on 0.6 of trials. Bearing in mind that an error cannot be repeated until it has been output a first time, 25% of those trials must be discounted (each stimulus was cued 4 times), leaving a maximum proportion of 0.75. For Lansdale and Laming (1995) and Laming (2021) the equivalent maximum (with 3 tests of each stimulus) is 0.67, and the observed proportions are 0.435 and 0.256. It is not just the fact of repetition, but the large proportion of trials that it engages. The fourth experiment, Ross and Bower (1981, Expt. 3) presents a different picture. The proportion of completely correct recalls was much higher, and most of the errors consisted of the retrieval of a single word from the wrong quartet.

**Table 5:** Proportions of each kind of error repeated in the four experiments, calculated with respect to the total number of trials

Category of error	Jones (1978)	Lansdale and Laming (1995)	Laming (2021)	Ross and Bower (1981, Expt. 3)
Answer (lag 0)	0.048	0.058	0.023	
Answer	0.143	0.082	0.084	
Yoked triple	0.055			0.005
Cued triple	0.114			
Yoked triple guess	0.038			
Yoked pair	0.088	0.106	0.053	0.016
Cued pair	0.061	0.129	0.069	
Yoked guess	0.052	0.060	0.027	
Single retrieval				0.084
Total	0.600	0.435	0.256	0.105

The repetition of previous errors is most prevalent when correct recall is difficult and participants are asked to guess. But those previous errors are available for repetition and must have been recorded in memory. Of course, I cannot speak to those response combinations that were not repeated, but I prefer the idea that every event is separately recorded in memory to the task of explaining why these particular events, and only these, were retained.

Historically, there have been other examples. Bartlett (1932) studied the retention of stories by asking participants for a written reproduction. Successive reproductions of “The War of the Ghosts” showed that changes from the original introduced in a first reproduction persisted through subsequent reproductions. “In a chain of reproductions obtained from a single individual, the general form, or outline, is remarkably persistent, once the first version has been given. ⋯ With frequent reproduction the form and items of remembered detail very quickly become stereotyped and thereafter suffer little change.” (Bartlett, 1932, p. 93). Bartlett’s study was replicated by Kay (1955) with participants making seven successive reproductions at intervals of a week and listening to the original again after each reproduction. Participants did not learn to correct the errors in their original reproductions. Instead, “The outstanding result was ⋯ each subject established an extraordinarily close relation between one reproduction and another.” (Kay, 1955, p. 93). The participants recalled chiefly what they had originally written and each reproduction rewrote that original reproduction in memory.

Mandler (1972, pp. 158–160; see also Hanawalt & Tarr, 1961) compared cued recall with recognition of a list of 60 words selected from 20 different categories. Recall was cued by the category labels. Intrusions on the recall test that subsequently appeared as lures in the recognition test were recognised as ‘Old’ 92%. This is to be compared with 97% for words originally presented and correctly recalled and only 57% for words presented but not recalled. The repetition of previous responses, whether correct or errors, appears to be widespread, and the re-analysis in Table 2 and Tables A and B in Review_tables.doc simply exhibits this phenomenon in detail. Many answers are recorded in memory and may be retrieved to provide the response(s) on some subsequent trial. Moreover, such retrievals are more potent than the original stimulus, a property that underlies the ‘generation effect’ and the ‘testing effect’ (Roediger & Karpicke, 2006; Slamecka & Graf, 1978).

Proposition 1 also speaks of an ‘ordered record’. Figures 10, 11, 12 and 13 show that the probability of repetition depends on how recently the original error was made and recency must also be represented in memory. In free recall experiments in which the participants have rehearsed out loud (Brodie & Murdock, 1977; Murdock & Metcalfe, 1978) it is possible to predict the ensuing sequence of recalls, not perfectly, but with significant success. Rehearsal follows a relatively simple pattern in which short sequences of words are recycled until the next stimulus word is presented (visually), whereafter rehearsal restarts, incorporating the latest stimulus. If that pattern of rehearsal is modelled and then run on after the signal to begin recall, it provides a good approximation to the sequence of recalls actually recorded (Laming, 2006, 2008). For rehearsal to repeatedly recycle previous rehearsals, those previous rehearsals have to be stored in memory in historic order.

### Correct recalls

Table 2 and Tables A and B in Review_tables.doc concern only the repetition of errors, but Figs. 3, 5, 7 and 9 demonstrate a related effect with correct recalls. A completely correct first recall increases the probability of a correct recall on the next test, and this enhancement extends to second and third recalls. It applies also to the repeated recall of answers (Figs. 3, 5, 7). While the repetition of a previous erroneous answer must be a retrieval from memory, the retrieval enters an additional copy of the answer in memory.

One alternative is that those stimuli that show increased recall on second test were learned more thoroughly on initial presentation. This is Estes’ (1960) idea of all-or-none learning. Estes presented his argument with several experiments of RTT design, one presentation of a stimulus–response pair for study followed by two tests. If the first test fails, the pair has not been retained in memory and the second test must also fail (correct guesses excepted). One such experiment (Estes et al., 1960) paired eight consonant triples with the digits 1–8 and showed a pattern of responses on repeated test very much like Fig. 9 (Ross & Bower, 1981, Expt. 3). This experiment was replicated by Jones (1962), but with four trials following the one presentation of the stimulus–response pairs. Jones’ (1962) results show a pattern very much like Fig. 3a (from Jones, 1978), including improvement on a fourth test following an initial failure (see Laming, 2019, Fig. 11). While enhanced attention to some stimuli may produce increased recall (and does; see Laming, 2021), the all-or-none hypothesis cannot stand.

### Reminiscence

The improvement on repeated testing accounts for the phenomenon of reminiscence. Ballard (1913) reported a seemingly counterintuitive finding: In the course of his employment as an HM Inspector of Schools, he would ask groups of schoolchildren to learn a poem with deliberately insufficient time allowed. The children were asked to write out as much as they could remember. Ballard would then return unexpectedly a few days later and ask the schoolchildren to write the poem out again. Scoring the number of whole lines reproduced without error that number increased on repeated test, over the score on initial test, by up to 20% (see Woodworth & Schlosberg, 1955, Fig. 25–6, p. 794). Since retention usually decreases with lapse of time, this increase seemed paradoxical, and was labelled ‘reminiscence’.

Reminiscence (more recently hypermnesia) has proved a fragile phenomenon, difficult to replicate (for reviews, see Payne, 1987; Erdelyi, 2010). But the principle that every event to which the participant attends is separately recorded in memory means that lines of poetry written during the initial test would have been recorded in memory and available for retrieval on a subsequent test. Now Ballard scored only the numbers of lines reproduced without error; this is like selecting completely correct recalls only in Fig. 3, etc. The second test, of course, showed an increased amount recalled (cf. Roediger & Thorpe, 1978). But the schoolchildren had no access to the poem in between whiles and the process has to be a martingale. So if Ballard had also examined lines that were reproduced incorrectly, he would surely have found his school children repeating their errors on the second test. In short, comparison with Figs. 3, 5, 7 and 9 makes reminiscence appear an artefact resulting from a biased selection of data.

2. The compilation of the record is automatic; while attention to a stimulus is at the participant’s disposal, the consequent entry into memory is not.

Figures 2, 4 and 6 plot the proportions of different kinds of error conditional on their presumed source. In view of the ‘generation’ and ‘testing’ effects (Roediger & Karpicke, 2006; Slamecka & Graf, 1978), it is perhaps plausible that sources that are themselves repetitions of previous errors should be recorded in memory, but what about Null sources comprising two or three independent guesses? Why should participants remember those? An alternative possibility is that repetitions of a Null source are no more than apparent, a combination of chance guesses.

Table 6 tabulates the number of trials (in the first three experiments) on which a Null source might have been matched completely, the number of trials on which such a complete match was observed, the chance probability of such a match and, finally, a normal deviate corresponding to the excess number of answers over expectation, calculated from the binomial distribution. While some of these answers might be chance matches, it is clear that most are true retrievals of Null sources, which must have been recorded in memory. So, why do participants retain combinations of two or three independent guesses? If entry into memory is automatic, that question is immediately answered.

**Table 6:** Answers retrieved from Null sources with a statistical evaluation

Experiment	No. of Null sources from which an answer might have been retrieved	Chance probability of a match	No. of answers retrieved from Null sources	Normal deviate
Jones (1978)	159	1/728	5	10.232
Lansdale and Laming (1995)	827	1/80	57	14.605
Laming (2021)	645	1/99	74	26.573

3. The retrieval of a potential response from memory is spontaneous; that retrieval becomes an overt response if it is compatible with the cue.

### Yoked recalls

There is a long-held tradition that learning occurs by the successive association of one idea with another (e.g. Ebbinghaus, 1885/1964, Ch. IX). Successive paired-associate trials, in which participants learn a list of stimulus–response pairs, giving a particular response to each different stimulus, were thought to strengthen such an association (e.g. Thorndike, 1913). More recently Anderson and Bower (1973), Brown et al., (2007), Lewandowsky and Murdock (1989), Lohnas et al. (2015), Polyn et al. (2009) and Raaijmakers and Shiffrin (1980), among others, have proposed extensive theories, based on the idea that the stimulus is instrumental in retrieving the response. These theories accommodate, though only approximately, a range of different experimental procedures. But this is no more than a habit of thought; it is not an established fact.

Analysis here shows that events on individual trials are recorded in memory, whence they may be retrieved to provide a response. So, successive paired-associate trials increase the pool of previous S–R pairs. Suppose that these pairs are retrieved spontaneously. If the pair *S*_*i*_–*R*_*i*_ comes to mind while *S*_*i*_ happens to be the cue, then *R*_*i*_ is produced as response. One can distinguish between *S*_*i*_ as the agent in procuring *R*_*i*_ as response, on the one hand, and spontaneous retrieval, on the other, only to the extent that experimental data permit. But the same probability of recall obtains on both scenarios (Laming, 2019, pp. 46–48). Experiments can estimate the probability of responding *R*_*i*_ to *S*_*i*_ as cue, but that estimate tells us nothing about how *R*_*i*_ is accessed.

But the incidences of yoked pairs and triples in Tables 2 and Tables A and B in Review_tables.doc speak powerfully to this issue. A yoked recall is a pair (or triple) sourced to some previous trial that does not contain the cue from the current trial. It follows that the current trial cue could have had no part in eliciting this particular (yoked) recall. The rule that applies equally to both cued and yoked recalls is simply that recall must be compatible with the trial cue. Recall from a source that contains the current trial cue is, ipso facto, compatible; but so also are yoked recalls from sources that do not contain the trial cue. The incidence of most categories of yoked recall is highly significant. Since in those cases the trial cue could not have been the agent of recall, retrieval must be spontaneous.

### Inverse recency

Are repeated combinations of errors retrieved as successions of single retrievals, component by component, or are they the product of a single retrieval, a triple or a pair (a ‘chunk’, Miller, 1956)? While it is common for the experimenter to analyse recalls in terms of individual components, there is no reason why memory should follow suit. The inverse recency exhibited by yoked pairs in Figs. 11 and 12 speaks powerfully to this question.

The lag–recall relations in Fig. 11 for yoked pairs on the one hand and for answers and cued pairs on the other are different; the difference is highly significant, this after allowing for the different levels of repetition (Laming, 2019, p. 22).^7^ Suppose the trial cue fails to elicit a retrieval, and the participant then selects a component at random and uses that component as cue to retrieve a partner from some previous answer. That is the very procedure that is assumed in the retrieval of cue pairs and, in consequence, their respective lag–recall relations should be the same. But, in fact, they are very different. It follows that yoked pairs are not retrieved through random selection of a cue; they must instead be retrieved as a pair. Suppose further that a complete answer is observed when a yoked pair or triple happens to match the trial cue; that is, the procedures for retrieving yoked pairs and complete answers are the same. In that case their respective lag-recall relations would also be the same. But, again, they are very different. It follows that complete answers do not result from a yoked pair matching the trial cue; they are the product of a single retrieval, a triple.

This argument requires two qualifications. First, the probabilistic nature of retrieval does not preclude some yoked pairs being the confluence of two single-component retrievals, or some answers being the confluence of a yoked pair matching the trial cue. All that can be said is that yoked pairs are *generally* retrieved as a pair and answers *generally* retrieved as a triple. Second, the argument that yoked pairs *are* retrieved as pairs could be inverted to argue that cued pairs are the consequence of a single retrieval matching the trial cue. I return to this issue below (Inverse recency, again).

### Conditional proportions of repetitions

The manner of retrieval set out above, and the qualification ‘generally’, bear on the relation of a repeated error to its source. Repetitions at lag 0 are instructive. If the trial cue happens to be one of the responses on the immediately preceding trial, the probability of an exact repetition is near 1 (Figs. 2, 4, 6) except when that preceding trial is Null, that is, comprising two or three independent guesses. Now those independent guesses might be separate entries in memory. In consequence, the probability of repeating all of them on the next trial is much reduced. For the same reason, the probability of retrieving an answer from a Null source is reduced, while the probability of repeating a complete answer is increased over other sources. Those other sources consist of a pair (cued or yoked) with some other single component that might be a separate entry in memory.

In free recall, after the first flush of recalls accessed by continuing the process of rehearsal (Laming, 2006, 2008), free recalls are found to include words already recalled, intrusions from previous lists and even from outside the experiment altogether (Diesfeldt, 2017; Howard & Kahana, 1999; Laming, 2012). How are these recalls obtained? Candidate words come to mind spontaneously and the problem for the participant is: is this, or is it not, a word that was presented in the list to be recalled? Not surprisingly, participants sometimes get it wrong. In the 1728 trials in the experiment by Ross and Bower there were 78 intrusions, of which 60 came from outside the experiment altogether. There were seven intrusions produced by two different participants; those participants had served in previous verbal experiments.

The idea of spontaneous retrieval is intuitive (see Zeigarnik, 1927; Woodworth, 1938, p. 51). It has long been known that participants tend to repeat their previous responses, errors as well as correct responses, with a frequency greater than chance. Thorndike (1931, Ch. 3) surveyed a large number of experiments of RTRTRT⋯ design, asking whether being told that a response was “Right” had a greater effect on subsequent responding than being told “Wrong”. Extracting sequences of responses to particular words over successive trials, Thorndike commented “Indeed the announcement of ‘Wrong’ in our experiment does not weaken the connection at all, so far as we can see. Rather there is more gain in strength from the occurrence of the response than there is weakening by the attachment of ‘Wrong’ to it.” (p. 45).

Welford (1968, pp. 296–300) pointed out that a common obstacle to learning a sequence of responses is errors made on the first few trials. Kay (1951) asked participants to learn to press a sequence of five Morse keys in a particular order, an order that had to be discovered by trial and error. There were five lights to provide feedback. When the next key in sequence was pressed, one of these lights extinguished and another came on in its place. If a wrong key was pressed, nothing happened, so that participants had always to find the correct next key before continuing. In spite of this forced correction, errors made on the first two or three trials persisted for many subsequent trials. Forbes (1988, p. 59) has reported a similar persistence of initial errors by a 2-year-old child.

If errors are prevented on the first few trials, learning is thereby expedited. Von Wright (1957) demonstrated this with human participants learning a 12 choice-point finger maze. He compared three groups: (a) learning from the outset by anticipation (average 24.25 trials to criterion); (b) four initial guided trials with advance information which choice is correct, thereafter learning by anticipation (average 10.45 trials to criterion); and (c) four initial forced trials, again thereafter learning by anticipation (average 18.80 trials to criterion).

### Retrieval as a function of lag

The question has long been asked (cf. Brown, 1958) is: how does retrieval fall away as lag increases? Figures 10, 11, 12 and 13 exhibit a pattern of spontaneous retrieval with respect to lag and, within each experiment, that pattern should be the same for all categories of retrieval. But retrievals are subject to constraints implicit in the experimental design. For example, a previous response combination might be retrieved entire, but if it does not contain the trial cue, it is ipso facto incorrect and not produced as a response. One must envisage, therefore, more retrievals than are recorded in the data. But retrievals are not instantaneous. In the second experiment by Lansdale and Laming (1995) the mean response latency for the first component of a completely correct recall was 5.28 s and the corresponding latency for a guess was 9.41 s (Laming, 2019, p. 29). Trials in the other experiments seem to have proceeded at a similar pace (Table 4).

Figures 10, 11, 12, 13 and 14 might appear to provide a wealth of data on that pattern of retrieval. But successive retrievals are not necessarily independent. The rehearsal data from Murdock and Metcalfe (1978) show a specific tendency to rehearse successive entries in the rehearsal sequence (Laming, 2006). Moreover, partial repetitions do not show the same lag characteristic as complete answers. Retrieval decreasing as lag increases has long been accepted, but yoked pairs in Fig. 6 (lags 0–7), 7 (lags 0–8) and 5 (lags 0–3) show a contrary relation. Finally, the divergence of correct responding in Figs. 3, 5, 7 and 9 points to the simultaneous retrieval of several previous responses, so disentangling the pattern of retrieval as a function of lag has to accommodate a possible multiplicity of sources.

### Inverse recency: again

An argument above (Inverse recency) showed that yoked pairs were generally retrieved as pairs and answers as triples. Suppose now that the response combination at lag *l* does not contain the trial cue. A retrieval from lag *l* might be either a complete answer or a cued pair or a yoked pair and these categories are mutually exclusive. If the retrieval is an answer or a cued pair, it would not show in Figs. 11 and 12, because it is incompatible with the trial cue. But a yoked pair meets no such constraint. The increasing prevalence of answers at short lags means that the retrieval of yoked pairs is correspondingly reduced and this does show in Figs. 11 and 12 as inverse recency.

Inverse recency continues to lag 7 (Fig. 11), not beyond, because, when individual stimuli are cued every nine trials, the response combination at lag 8 contains a component (the lag 8 trial cue) from the stimulus addressed on the current trial. Retrieval of a yoked pair from lag 8 would add two components from the stimulus addressed on the current trial and would be read as an answer. In Fig. 12, where stimuli were cued every ten trials, the inverse recency extends to lag 8, again because a yoked pair from lag 9 would be read as an answer. Thereafter, yoked pairs appear to exhibit the same decreasing lag-recency (see Figs. 11, 12) as other categories of repetition. At longer lags the probability of retrieving an answer decreases to the point that suppression of yoked pairs is no longer apparent.

Comparing the lag–recall relations of yoked pairs and cued pairs, it might appear that if yoked pairs are retrieved as pairs, then cued pairs have to be the consequence of a single retrieval matching the trial cue. But the difference between the two lag-recall relations subsists chiefly in the negative recency shown by yoked pairs. That negative recency is attributed above to the prevalence of answers rather than cued or yoked pairs in retrievals at short lags. The retrieval of cued pairs would, of course, be reduced in the same way as yoked pairs, but, since a cued pair would be incompatible with the trial cue, it does not show (as negative recency) in Fig. 11.

### *Ross* and Bower (1981*, Expt. 3)*

While the repetition of errors (Fig. 13) is similar to the pattern in the visual experiments, the recall of correct and partially correct responses shows a much slower (1/20) rate of decrease as lag increases. Possibly that much slower rate resulted from the participants ‘encoding’ each quartet on presentation. The quartets in this experiment were selected at random from the pool used in a previous experiment; and the quartets in that previous experiment (Ross & Bower, 1981, Expt. 2) were deliberately grouped around some sense impression or (in Expt. 1) around a scenario. So, assuming these participants had served in those previous experiments, they would have been primed to find some overarching theme (‘encoding’, Craik & Tulving, 1975) in the 10 s for which each quartet was visible. Thereafter, it looks as though on about half the trials the cue word enabled the encoding to be identified and about half of those identified encodings enabled the complete quartet to be retrieved. Errors, of course, are subject to no such encoding and show a similar pattern to the visual experiments.

Encoding might also account for this finding. In most cases the set of words recalled on the second test was the same as that on the first. But in a proportion of cases, exceeding 10%, the cue and one or two words recalled on the first test *did not include* the cue word that was presented on the second test (Triple and double retrievals in Fig. 8). Nevertheless, that second test reproduced all the words recalled on the first. How was the record of that first test identified? If the second test cue identified the encoding, that encoding might then have accessed the original stimulus and so all of the words previously recalled.

Finally, the much slower rate of decrease of accessibility for correct recalls suggests an answer to a difficult problem. The rates of decrease in Figs. 10, 11, 12 and 13 are much too rapid for the material in question (errors) to survive into the long term. If, however, the material is ‘encoded’, the much slower rate in Fig. 14 makes survival plausible, especially when after a second test there appears to be no further decrease. So, trivial entries in memory quickly become inaccessible, but other kinds of entry seem much more durable and maybe form the accessible content of memory in the long term.

## Discussion

This article has reviewed the results from four ‘fragmentation’ experiments—not the already published results, but results from a reanalysis of the original data, looking for repetitions of errors. Participants were not asked to recall their previous errors. The repetitions in Figs. 10, 11, 12 and 13 are therefore incidental observations, uncontaminated by participants’ strategies. Repetition of errors happens naturally in the course of recall and constitutes a novel genre of findings. This review has spelled out the implications for our understanding of memory over the short term. The discussion below reviews each category of findings in turn.

The first three experiments are similar in material, design and results. Ross and Bower (1981, Expt. 3) is different. It used verbal material and is particularly valuable for that reason. The stimulus quartets were tested twice only; a complete set of tests using all four words would have been especially informative. But participants were not asked to guess; and probabilities of guessing could not have been calculated, anyway. The nine or ten values on three or four independent attributes in the first three experiments enabled the identification of repetitions with high reliability. Calculation of chance probabilities is essential to the evaluation presented here. But this is a feature of the experimental design, not of memory itself; there is no reason to suppose that memory functioned differently in consequence.

### Repetition of errors

Many combinations of responses are repetitions of combinations on previous (incorrect) trials. The probability of this kind of match occurring by chance can be calculated and is tiny in relation to the numbers of repetitions. It follows that most of those repeated errors must have been recorded in memory. Participants in the first three experiments were asked to guess if they could not remember; many of their guesses turned out to be retrievals from memory. Various explanations might be proposed why these particular retrievals were repeated, but the simplest is:

Every event (a stimulus or a response or just a retrieval) to which the participant attends is separately recorded in memory, creating an ordered record of those events that have engaged the participant’s attention.

Oberauer et al. (2018) and 15 others actively engaged in the study of short-term/working memory have published a list of experimental phenomena (benchmark findings) that, in their collective judgement, any theory should strive to explain. Their list includes several findings concerning errors in recall:

Confusions of target item with other items in the memory set (e.g. Henson et al., 1996).

Serial position effects on types of error (intrusions, omissions and repetitions of an item already recalled) in serial recall. (e.g. Henson et al., 1996).

Intrusions from previous memory sets (e.g. Drewnowski & Murdock, 1980; Fischer-Baum & McCloskey, 2015; Quinlan et al., 2015).

Ranschburg effect in serial recall (e.g. Crowder, 1968; Henson, 1998).

There is, however, nothing in their collective list about the repetition of errors. This review has revealed several findings, highly significant, that need to be added to Oberauer et al.’s list.

### Correct recalls

The repetition of previous (erroneous) response combinations applies equally to correct recalls. If a stimulus is tested repeatedly, a completely correct recall on first test enters an additional copy of the stimulus in memory and increases the probability of a correct recall on the next test. This enhancement extends to third and fourth tests. If the participants have no intermediate access to the stimuli, the process is a martingale; but the probabilities of correct recall following different prior sequences diverge (Figs. 3, 5, 7, 9). This provides explanations for ‘all-or-none’ learning (Estes, 1960), for the fragmentation hypothesis (Jones, 1976; see Laming, 2019) and for reminiscence (Ballard, 1913).

### Storage in memory

Some of the repeated errors were traced to Null sources comprised of 2 or 3 independent guesses. While such matches could simply result from chance, analysis of the frequencies of such matches (Table 6) showed those repetitions to be highly significant. Again, various explanations might be proposed for retaining Null responses in memory, but the simplest is:

The compilation of the record is automatic; while attention to a stimulus is at the participant’s disposal, the consequent entry into memory is not.

This would explain, in addition, how it is that we are able to remember many events in our everyday lives without there being any conscious effort or intention to retain them.

### Retrieval

Many of the repeated error combinations have a component in common with the trial cue, but some (yoked pairs and triples) do not. The trial cue could not have been instrumental in eliciting those yoked repetitions. Various explanations might be proposed, but the simplest is:

The retrieval of a potential response from memory is spontaneous; that retrieval becomes an overt response if it is compatible with the cue.

The rule that applies equally to both cued and yoked recalls is simply that recall must be compatible with the trial cue. If a retrieval contains the trial cue, of course it is compatible; but so also is a yoked recall from a source that does not contain the trial cue.

### The relation between lag and recall

The analysis that identifies a trial as a repetition in the first place also identifies the lag between repetition and source. So the ‘fragmentation’ experiments reveal not only frequencies of repetitions, but also the pattern of retrievals with respect lag in the trial sequence (Figs. 10, 11, 12, 13). A model for these experiments needs a list of preceding trials in inverse order, with lag 0 at its head (cp. Laming, 2009), together with an algorithm for selecting preceding trials for retrieval. The question above all others is: how does retrieval fall away as lag increases? Figures 10, 11, 12 and 13 might appear to provide a rich lode of data to answer this question, but I think that answer would be premature.

Retention in the short term has been investigated in particular by the Brown–Peterson procedure: material presented at time 0 is recalled at some later time *t,* the interval (0, *t*) being occupied with a demanding task that is thought to preclude rehearsal (Peterson & Peterson, 1959; see Laming, 1992 for a review). Brown (1958) reported three experiments in which interpolated material, not for recall, depressed the recall of other material presented previously.^8^ Brown spoke of ‘trace decay’, proposing that material in memory decayed to the point that it could no longer be retrieved. But if ‘decay’ really means decay, it is a non-starter, because we are all able to remember many things that happened a long time ago. How are those memories accessible long after the point in time at which they should have decayed? This problem has been universally ignored (e.g. Atkinson & Shiffrin, 1968; Broadbent, 1958; Miller, 1956). Psychologists have modelled their laboratory experiments without regard to the faculty that those experiments are ostensibly studying (cp. Hintzman, 2011).

Brown (1958) was arguing against the then fashionable view that failure to recall was due to interference from previous entries in memory (Underwood, 1957; Melton, 1963; and more recently Lewandowsky et al., 2009; Nairne, 2002; Oberauer & Kliegl, 2006). This poses the question: How can one entry in memory interfere with another? If participants are taught two or more different responses to the same stimuli (e.g. Barnes & Underwood, 1959), of course there is confusion leading to a loss of recall. But that is not generally the case. The work of Keppel and Underwood (1962) implies that the mere presence of previous entries in memory (proactive interference) is sufficient and Watkins and Watkins (1975) introduced the notion of ‘Cue overload’:

“The principle states that the efficiency of a functional retrieval cue in effecting recall of an item declines as the number of items it subsumes increases.” (Watkins & Watkins, 1975, p. 443)

But how does the prior presentation of extraneous material interact with that which is to be recalled?

Both Brown’s ‘trace decay’ and the idea of interference assume that the cue for recall is in some away directly involved in retrieval. This is no more than assumption. Prior to Brown (1958) the predominant study was learning, especially paired associates, where it was natural to think of an increasing association between stimulus and response, gradually strengthened over a succession of test trials. But such an improvement in recall could equally be modelled as an accumulation of discrete records in memory, as in Figs. 3, 5, 7 and 9. The issue here is not the state of nature, but how that state is modelled. Existing data are equally compatible with spontaneous retrieval, with responses selected after retrieval according as they are compatible with the cue.

Spontaneous retrieval realises the functions attributed both to trace-decay and to interference. The period of the experimental test is occupied with whatever material the experimenter presents or the participant rehearses. Since we can retrieve only one idea at a time, there has to be some selection governing what is retrieved and it happens to be weighted towards the most recent (Figs. 10, 11, 12 and 13 above). An accessibility decreasing as lag increases ensures that material becomes inaccessible after a period of time. This is realised functionally by a decaying trace, except that it is the accessibility that falls off, not the trace in memory, which can survive into the long term. (But there is still a problem here that I return to below; see Ross & Bower, 1981, Expt. 3).

When recall is requested, the mechanics of memory select from previous traces according to their decreasing accessibility. For example, the Brown–Peterson experiments by Keppel and Underwood (1962) can be satisfactorily modelled with a reciprocal weighting function (Laming, 1992, pp. 1348–9). The accessibility of a particular trace falls off and other material is recalled in its place. Bearing in mind that a successful recall writes the stimulus again in memory, this is also Thorndike’s (1913) Law of Disuse. One might say that the presentation of additional material ‘interferes’ with everything else in memory by pushing it further into the past (Laming, 2009). One might also say that the presentation of additional material makes everything else in memory less accessible—a liberal interpretation of ‘decay’ (Cowan et al., 1997). But the ‘might’s above merely obscure the idea of spontaneous retrieval.

### Ross and Bower (1981, Expt. 3)

While spontaneous retrieval can accommodate the functions ascribed to both ‘trace decay’ and interference, it does not itself resolve the problem of how material can be accessed long after the point in time at which it should have decayed beyond retrieval. The correct recalls in Fig. 14 show a rate of loss of accessibility one twentieth of that for the errors in Fig. 13. Moreover, after a second trial, there appears to be no further decrease, not within the time span of Ross and Bower’s experiment. I conjectured (above) that the participants ‘encoded’ each quartet on presentation, meaning by ‘encoded’ the selection of some overarching theme to characterise each quartet. Thereafter, that encoding might have provided an alternative route to the stimulus. It may be that long-term memory preserves different categories of memories, more durable than a simple error of recall.

### Models of memory

Since Norman (1970), at least, there has been an explosion of models of short-term memory—to be precise, models for laboratory experiments that track retention over short periods of time. The number and variety of models is itself testimony to the paucity of empirical information how memory actually works; conjectures have flourished with little constraint. Ross and Bower (1981), for example, tested three different models of the structure of a memory trace, of which the fragmentation hypothesis was one. Such a test depends on the assumption that different cues address the same trace in memory, and the sources identified above show that that is often not so. Indeed, if it be assumed that the cues addressing a particular stimulus elicit their responses from a common source, the repetition of errors generates data that strongly suggest all-or-none learning (Estes, 1960) and the fragmentation hypothesis without those ideas having any relevance to the state of nature. Joensen et al. (2019) have recently reopened the issue of the fragmentation hypothesis. Their triples were presented as three stimulus pairs on separate trials, with an assumption that the three separate pairs would be integrated into a single engram. Not only might the trial cue address one pair alone, it might well address, instead, a previous recall (see Laming, 2021). In short, experimental tests of such ideas are unreliable.

## Summary

Models of memory per se are outside the scope of this review. It has instead presented a novel genre of analysis and results that provides significant new insights into the mechanics of memory. Most of those insights can be summed up in three propositions:

1. Every event (a stimulus or a response or just a retrieval) to which the participant attends is separately recorded in memory, creating an ordered record of those events that have engaged the participant’s attention.
2. The compilation of the record is automatic; while attention to a stimulus is at the participant’s disposal, the consequent entry into memory is not.
3. The retrieval of a potential response from memory is spontaneous; that retrieval becomes an overt response if it is compatible with the cue.

In addition, this review has examined some particular assumptions that might, or might not, feature in a model. The most important is the notion of a guess. When participants guess, they do not select at random from the available alternatives—they retrieve their ‘guesses’ from memory. Calculations based on random guessing are suspect.

The tracing of a repetition to its source is, in effect, the observation of an individual retrieval, so that the pattern of activity in short-term memory can be observed directly. The ideas of ‘trace-decay’ and of interference as general explanations of loss of accessibility with lapse of time become redundant. The functions accomplished by both of those ideas are subsumed in spontaneous retrieval. Likewise, the idea that the trial cue is a direct agent in retrieval. That idea is implicit in both ‘trace-decay’ and interference, but is incompatible with yoked recalls. It must be abandoned.

Finally, two ideas that merit further exploration: first, tracking the repetition of errors in recall has revealed an automatic storage in memory and a pattern of retrieval biased toward the most recent entries. There is no reason why this pattern should be restricted to errors; it is simply that the small probability of matching a previous error by chance enables retrievals from memory to be traced. Repeated storage and retrieval enables the assembly of more complex ideas than can ordinarily be contained in a single retrieval. It constitutes a system for holding information in mind and working on it– a function commonly labelled ‘working memory’.

Second, the rate at which accessibility is lost means that the material recorded in Figs. 10, 11, 12 and 13 could not survive into the long term. But the much slower rate of loss by correct recalls in Fig. 14 suggests that different categories of memories lose accessibility at different rates, and it may be that long-term memory preserves different categories of content.

### Supplementary Information

Below is the link to the electronic supplementary material.

Supplementary file1 (DOC 133 KB)

## Figures and Tables

**Fig. 15:**
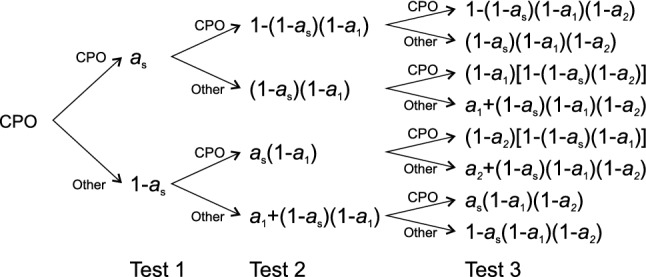
Formulae for probabilities of CPO recall, conditional on all preceding recalls

## Data Availability

The original data is in my possession and will be made available on reasonable request. The code needed to reanalyse that data is also in my possession and will again be made available if required, but note that re-running data and code in a c suite will not be straightforward. The results of interim analyses will also be made available on reasonable request; these are chiefly the results presented in the figures.

## Appendix

### Model for divergent patterns of responding

The model implements this rule: The *n*th trial looks at entries in memory of the original stimulus and of the (*n* − 1) intervening trials that may be either correct or wrong. The recall on trial *n* is correct if both one of the correct entries (including the stimulus) is retrieved and none of the incorrect entries. If any of the incorrect entries is retrieved, the recall is incorrect.

Let *a*_s_ be the accessibility (probability of retrieval) of the stimulus; the stimulus is assumed to be sufficiently remote from the trials that *a*_s_ shows no material variation through successive tests. Let *a*_1_ be the accessibility of the record of the preceding test, that is, of Test 1 on trial 2 and Test 2 on trial 3. Likewise, let *a*_2_ be the accessibility of Test 1 on trial 3. Then the conditional probabilities of recall, conditional on the outcomes of all preceding tests, are as shown in Fig. 15. The probabilities of CPO recalls map directly onto Fig. 5.

These model probabilities are conditional on the preceding sequence of recalls and failures to recall. So, the probability of a correct recall on Test 1 is, of course, *a*_s_. The probability of a correct recall on Test 2, given a correct recall on Test 1, is equal to the probability that either the original stimulus or the record of Test 1 is retrieved; and the probability of an error is equal to the probability that neither of these entries is retrieved, that is, (1 – *a*_s_)(1 – *a*_1_). The probability of an error on Test 3, to take another example, given a error on Test 1 and a correct recall on Test 2, is equal to the sum of the probability of a retrieval of the error on Test 1 (*a*_2_, because there is Test 2 intervening) and the probability that neither the stimulus nor either of the preceding tests is retrieved; that sum is *a*_2_ + (1 – *a*_s_)(1 – *a*_1_)(1 – *a*_2_), and so on.

The model is evaluated by comparing the absolute probability of each sequence of correct recalls and errors with the numbers actually occurring. For example, the probability of three successive errors is [(1 – *a*_s_)][*a*_1_ + (1 – *a*_s_)(1 – *a*_1_)][1 – *a*_s_(1 – *a*_1_)(1 – *a*_2_)]. This is the absolute probability of one of eight different sequences of three successive responses. The comparison yields a *χ*^2^ statistic with four degrees of freedom—eight subdivisions of the total less one, less a further three in respect of the three free parameters, *a*_s_, *a*_1_ and *a*_2_.

## Footnotes

1 This experiment was conducted in 1983–84 with the resources and facilities then available.

2 I thank Brian Ross for photocopies of the original participants’ response sheets. His data have been re-analysed *ab intio*.

3 A sequence of random variables *X*_1_, *X*_2_, *X*_*n*_ is a martingale if the expectation of *X*_*n*+1_ = *X*_*n*_. An immediate consequence is that the variance of the variables *X*_*n*_ is continually increasing. Since the proportions in Fig. 3 are constrained to the interval (0,1), each sequence of probability of recall will ultimately diverge to either 0 or 1.

4 Modified to accommodate four trials.

5 Repetitions of previous answers (and other errors too) exhibit different lag-recall curves depending on whether the presumed source contains a correct recall or is Null (Lansdale & Laming, 1995, p. 56). Ideally, the argument below should be presented twice, first with respect to sources that themselves contain recalls and a second time with respect to Null sources only. But this materially weakens the statistical argument by dilution of the data.

6 This inverse recency is, to my mind, distinct from the negative recency that is observed when a final free recall is requested following a series of ordinary free recall tests; Craik (1970), Kuhn, Lohnas & Kahana, (2018).

7 The corresponding comparisons in Fig. 12 are equally significant.

8 Ricker et al. (2016) have recently reviewed the impact of Brown’s (1958) paper.
