# Supplementary material for: The repetition of errors in recall: a review of four ‘fragmentation’ experiments
Source: Psychol Res. 2022 Mar 14;86(6):1699–724. doi: 10.1007/s00426-021-01598-z (PMC9363359; doi:10.1007/s00426-021-01598-z)
Supplement: Supplementary file 1 — Supplementary file1 (DOC 133 KB) [file 426_2021_1598_MOESM1_ESM.doc]

Table A. Incidences of different categories of repeated errors in the experiment by Lansdale and Laming (1995), with a statistical assessment of each category.

| Cue attribute | Response attribute(s) | Total trials | Number observed. | 95% confidence interval | Proportion | Chance expectation | Chi Square | Significance. |
| --- | --- | --- | --- | --- | --- | --- | --- | --- |
| Aggregate Lag 0 answer fragments | |  |  |  |  |  |  |  |
| C | PO | 140 | 84 | ‡ , 3.746 | 0.600 | 1.750 | 62.567† | 0.000 |
| P | CO | 164 | 92 | ‡ , 4.139 | 0.561 | 2.050 | 63.220† | 0.000 |
| O | CP | 128 | 77 | ‡ , 3.511 | 0.602 | 1.600 | 59.985† | 0.000 |
| Aggregate Lag > 0 answer fragments | |  |  |  |  |  |  |  |
| C | PO | 724 | 106 | 6.420, 20.680 | 0.146 | 13.55 | 645.8946 | 0.000 |
| P | CO | 735 | 108 | 6.989, 21.636 | 0.147 | 14.3125 | 628.7287 | 0.000 |
| O | CP | 795 | 143 | 7.678, 22.797 | 0.180 | 15.2375 | 1097.4194 | 0.000 |
| Aggregate cue pairs | |  |  |  |  |  |  |  |
| C | P | 430 | 73 | 51.353, 79.997 | 0.170 | 65.675 | 1.005 | 0.316 |
| C | O | 386 | 149 | 43.117, 69.933 | 0.386 | 56.525 | 182.733 | 0.000 |
| P | C | 489 | 64 | 63.260, 94.379 | 0.131 | 78.819 | 3.485 | 0.062 |
| P | O | 461 | 54 | 57.557, 87.518 | 0.117 | 72.538 | 5.883 | 0.015 |
| O | C | 387 | 154 | 43.744, 70.646 | 0.398 | 57.195 | 198.964 | 0.000 |
| O | P | 437 | 71 | 55.404, 84.838 | 0.162 | 70.121 | 0.014 | 0.907 |
| Aggregate yoked pairs | |  |  |  |  |  |  |  |
| C | PO | 732 | 109 | 79.833, 115.180 | 0.149 | 97.507 | 1.625 | 0.202 |
| P | CO | 889 | 263 | 83.252, 119.804 | 0.296 | 101.528 | 299.861 | 0.000 |
| O | CP | 709 | 93 | 75.394, 109.867 | 0.131 | 92.630 | 0.002 | 0.966 |
| Aggregate yoked guesses | |  |  |  |  |  |  |  |
| C | PO | 764 | 63 | 44.327, 73.113 | 0.082 | 58.72 | 0.3397 | 0.560 |
| P | CO | 900 | 147 | 44.321, 73.224 | 0.163 | 58.7727 | 143.1824 | 0.000 |
| O | CP | 731 | 54 | 45.057, 73.991 | 0.074 | 59.5238 | 0.56 | 0.454 |

‡ The conventional approximation to the lower limit of the 95% confidence interval fails when the chance expectation is very small.

^†^ Normal deviate from binomial test with probability 1/99.

Table B. Incidences of different categories of repeated errors in the recall of magazine advertisements in Laming (2020), with a statistical assessment of each category.

| Cue attribute | Response attribute(s) | Total trials | Number observed | | | | | 95% confidence interval | | | | | | | | | Proportion | Chance expectation | Chi Square | Significance |
| --- | --- | --- | --- | --- | --- | --- | --- | --- | --- | --- | --- | --- | --- | --- | --- | --- | --- | --- | --- | --- |
| Lag 0 answer fragments | | | |  | | | | | |  | | | | |  | | |  |  |  |
| B | PS | 90 | 44 | | | | | ‡, 2.778 | | | | | | | | | 0.489 | 0.909 | 45.196^†^ | 0.000 |
| P | BS | 89 | 37 | | | | | ‡, 2.757 | | | | | | | | | 0.416 | 0.899 | 38.075^†^ | 0.000 |
| S | BP | 107 | 56 | | | | | ‡, 3.119 | | | | | | | | | 0.523 | 1.081 | 52.821^†^ | 0.000 |
| Lag > 0 answer fragments | | | | |  | | | | | | | | |  | |  | |  |  |  |
| B | PS | 581 | 172 | | | | | 2.444, 13.379 | | | | | | | | | 0.296 | 7.9118 | 3459.72 | 0.000 |
| P | BS | 531 | 146 | | | | | 2.132, 12.721 | | | | | | | | | 0.275 | 7.4265 | 2631.3 | 0.000 |
| S | BP | 609 | 177 | | | | | 2.923, 14.334 | | | | | | | | | 0.291 | 8.6286 | 3345.53 | 0.000 |
| cued pairs | | | | | |  | | | | |  | | | | | |  |  |  |  |
| B | P | 304 | 73 | | | | | 29.184, 52.087 | | | | | | | | | 0.240 | 40.6352 | 30.6844 | 0.000 |
| B | S | 322 | 37 | | | | | 30.588, 53.981 | | | | | | | | | 0.115 | 42.2842 | 0.7841 | 0.376 |
| P | B | 294 | 65 | | | | | 28.448, 51.091 | | | | | | | | | 0.221 | 39.7694 | 19.0774 | 0.000 |
| P | S | 273 | 80 | | | | | 25.114, 46.665 | | | | | | | | | 0.293 | 35.8895 | 64.3765 | 0.000 |
| S | B | 339 | 44 | | | | | 33.598, 57.889 | | | | | | | | | 0.130 | 45.7437 | 0.0792 | 0.778 |
| S | P | 345 | 105 | | | | | 33.998, 58.393 | | | | | | | | | 0.304 | 46.1954 | 89.2889 | 0.000 |
| yoked pairs | | | | | | |  | | | | |  | | | | |  |  |  |  |
| B | PS | 639 | 116 | | | | | 44.679, 72.857 | | | | | | | | | 0.182 | 58.7678 | 63.3878 | 0.000 |
| P | BS | 503 | 62 | | | | | 38.112, 64.326 | | | | | | | | | 0.123 | 51.2192 | 2.5988 | 0.107 |
| S | BP | 657 | 132 | | | | | 52.759, 82.807 | | | | | | | | | 0.201 | 67.7829 | 70.1813 | 0.000 |
| yoked guesses | | | | | | | | |  | | | |  | | | |  |  |  |  |
| B | PS | 639 | 63 | | | | | 32.142, 57.346 | | | | | | | | | 0.099 | 44.7439 | 0.005 | 0.005 |
| P | BS | 568 | 42 | | | | | 31.194, 56.019 | | | | | | | | | 0.074 | 43.6063 | 0.800 | 0.800 |
| S | BP | 649 | 54 | | | | | 30.731, 55.521 | | | | | | | | | 0.083 | 43.126 | 0.086 | 0.086 |

‡ The conventional approximation to the lower limit of the 95% confidence interval fails when the chance expectation is very small.

^†^ Normal deviate from binomial test with probability 1/99.
